# Exosomes in ovarian cancer ascites promote epithelial–mesenchymal transition of ovarian cancer cells by delivery of miR-6780b-5p

**DOI:** 10.1038/s41419-021-03490-5

**Published:** 2021-02-24

**Authors:** Jing Cai, Lanqing Gong, Guodong Li, Jing Guo, Xiaoqing Yi, Zehua Wang

**Affiliations:** 1 Department of Obstetrics and Gynecology, Union Hospital, Tongji Medical College, Huazhong University of Science and Technology, Wuhan, 430022 China; 2 Cancer Research Institute, Tongji Hospital, Huazhong University of Science and Technology, Wuhan, 430022 China

**Keywords:** Cancer microenvironment, Ovarian cancer

## Abstract

The poor prognosis of ovarian cancer is mainly due to metastasis, and the specific mechanism underlying ovarian cancer metastasis is not clear. Ascites-derived exosomes (ADEs) play an important role in the progression of ovarian cancer, but the mechanism is unknown. Here, we found that ADEs promoted ovarian cancer metastasis not only in vitro but also in vivo. This promotive function was based on epithelial–mesenchymal transition (EMT) of ovarian cancer cells. Bioinformatics analysis of RNA sequencing microarray data indicated that miR-6780b-5p may be the key microRNA (miRNA) in ADEs that facilitates cancer metastasis. Moreover, the expression of exosomal miR-6780b-5p correlated with tumor metastasis in ovarian cancer patients. miR-6780b-5p overexpression promoted and miR-6780b-5p downregulation suppressed EMT of ovarian cancer cells. These results suggest that ADEs transfer miR-6780b-5p to ovarian cancer cells, promoting EMT and finally facilitating ovarian cancer metastasis.

## Introduction

Ovarian cancer has the highest mortality among gynecologic malignancies^1^. Although the treatment of ovarian cancer has improved in recent years, the five-year survival rate of ovarian cancer patients, which has remained at approximately 40%, has not improved significantly in the last twenty years—the survival rate in the last five years was only 41.8%^2^. Metastasis, nonspecific symptoms at early tumor stages and chemoresistance may be the main reasons for the poor prognosis of ovarian cancer^3,4^. Ovarian cancer patients generally manifest symptoms only after progression to an advanced stage, and only a few women are diagnosed before the tumor metastasizes to the peritoneal cavity^3^. More than 70% of ovarian cancer patients are at a late stage with peritoneal metastasis and are diagnosed with advanced-stage disease, resulting in a high risk of death^5^. Thus, it is imperative to illustrate the underlying molecular mechanisms behind tumor metastasis and guide the therapeutic strategy of ovarian cancer to improve prognosis. Implantation metastasis to the omentum and peritoneal organs is the most common form of ovarian cancer metastasis^6^. Peritoneal metastasis is often accompanied by the accumulation of malignant ascites, while the formation of omental metastasis with ascites accelerates tumor progression^3,7^. However, the facilitative role of ascites in tumor metastasis is still unclear. Therefore, it is important to determine the mechanism by which ascites promotes ovarian cancer metastasis.

Exosomes are extracellular vesicles with diameters of 50–140 nm that can carry small molecules, such as nucleic acids, proteins, and other bioactive molecules, to communicate between cells^8^. Exosomes secreted by tumor cells can promote tumor growth, metastasis, and angiogenesis and participate in the drug resistance of tumor cells^9,10^. High levels of exosomes have been found in the culture supernatant of ovarian cancer cells and ascites from ovarian cancer patients. Ascites-derived exosomes (ADEs) from ovarian cancer patients were found to be significantly correlated with tumor load, invasion, and short survival time^11^. Exosomes from ovarian cancer patients were also found to promote interstitial barrier clearance by peritoneal interstitial cells^12^. These results suggest that ADEs play an important role in the metastasis of ovarian cancer. Exosomal microRNAs (miRNAs) play an important role in the biological actions of ovarian cancer because these molecules are specific and closely related to the cell type and state^13^. For example, miR-6126 acts as a tumor suppressor by directly targeting integrin β1 and inhibits ovarian cancer cell metastasis^14^, and miR-6086 inhibits ovarian cancer angiogenesis^15^, while miR-21–3p, miR-125 b-5p, miR-181 d-5p and miRNA-205 in ovarian cancer exosomes promote tumor invasion and metastasis^16,17^. The serum miRNA profiles of patients with ovarian carcinoma constituted a promising diagnostic biomarker for ovarian cancer in a recent study^18^. Therefore, identification of the key miRNAs in ADEs is expected to contribute to clarifying the mechanism underlying ovarian cancer metastasis and help to identify therapeutic targets for ovarian cancer.

Cancer metastasis involves multiple steps, including the epithelial–mesenchymal transition (EMT)^19^. The conversion of an epithelial cell to a mesenchymal cell plays a key role in the invasion and metastasis of ovarian cancer^20^. In the present study, in vitro and in vivo experiments were conducted to verify the promotive effect of ADEs on ovarian cancer metastasis. The role of ADEs was validated in cancer cell lines and primary tumor cells. EMT markers were detected to determine whether ADEs affect EMT in ovarian cancer. Bioinformatics analysis of RNA sequencing microarray data indicated that miR-6780b-5p may be the key miRNA in ADEs that facilitates ovarian cancer metastasis. Moreover, the correlation of miR-6780b-5p with tumor metastasis was validated in ascites from ovarian cancer patients. Tumor cell migration and EMT markers were evaluated after miR-6780b-5p expression was regulated by transfection with an agomir or antagomir. We demonstrated the effects of miR-6780b-5p in ADEs as promoting EMT of ovarian cancer cells and finally facilitating ovarian cancer metastasis.

## Materials and methods

### Ascites specimens and primary cells

All ascites specimens of patients with ovarian cancer were collected from patients who underwent surgery for the first time at the Union Hospital of Tongji Medical College, Huazhong University of Science and Technology from October 2014 to October 2016, while cancer tissues that were surgically removed from two patients with high-grade serous ovarian carcinoma (HGSOC) were used for the culture of primary tumor cells. No patients had received radiotherapy or chemotherapy before the surgery, and all were pathologically diagnosed with ovarian cancer. The pathological characteristics of ovarian cancer patients whose ascites were collected are listed in Table 1. Fresh ascites was centrifuged at 300 × *g* for 10 min to remove cell components, and the cell components were collected and stored at −80 °C. Then, the supernatant was used for exosome extraction or stored at −80 °C. The pathological characteristics of ovarian cancer patients whose tissues were used for primary tumor cell culture are listed in Table 2. Primary tumor cells were extracted using the collagenase treatment method as described previously^21^ and cultured in DMEM/F12 medium supplemented with 10% fetal bovine serum (FBS, Gibco). All primary tumor cells were used for a maximum of three passages as previously described^22^. All procedures were conducted with the approval of the Ethics Committee of Tongji Medical College, Huazhong University of Science and Technology. Patient consent was obtained before the start of the study.

**Table 1:** The clinicopathological features of 27 ovarian cancer patients.

ID	Age (year)	FIGO^1^ stage	Pathological type	CA125 U/ml	Tumor size	Peritoneal metastasis	Relative miR-6780b-5p level
**#1**	60	IIC	Clear cell carcinoma	351.2	11.3 cm×11.3 cm×10.1 cm	No	45.88
**#2**	61	IIIC	High-grade serous carcinoma	>1000	10 cm×10 cm×8 cm	Yes	81.63
**#3**	51	IIIC	High-grade serous carcinoma	>1000	3.1 cm×2.2 cm	Yes	482.18
**#4**	37	IC	Mucinous cystadenocarcinoma	92.4	19 cm×14 cm×4 cm	No	1.00
**#5**	40	IV	High-grade serous carcinoma	600	8.1 cm×5.3 cm×4.6 cm, 6.3 cm×5.0 cm×4.6 cm	No	4.87
**#6**	72	IIB	High-grade serous carcinoma	>1000	3 cm×3 cm	No	55.78
**#7**	43	IIC	High-grade serous carcinoma	1475	8.4 cm×10 cm×7.9 cm	Yes	149.93
**#8**	23	IC	Borderline serous tumor	>1000	7.5 cm×6.7 cm×4.4 cm	No	7.01
**#9**	42	IV	High-grade serous carcinoma	>600	30 cm×25 cm	Yes	390.38
**#10**	24	IIIC	Borderline serous tumor	NA	7.3 cm×6.1 cm,8.8 cm×6.4 cm	Yes	275.34
**#11**	45	IIIC	High-grade serous carcinoma	850.8	15 cm×15 cm	Yes	49.54
**#12**	45	IIIB	High-grade serous carcinoma	>1000	6 cm×5 cm	Yes	25.03
**#13**	59	IIIC	High-grade serous carcinoma	>1000	7.6 cm×6.9 cm×6.3 cm	Yes	2.88
**#14**	52	IC	Clear cell carcinoma	4042.5	16.1 cm×12.7 cm×13.6 cm	No	6.23
**#15**	52	IC	Follicular cell tumor	>1000	13.0 cm×15.0 cm	No	65.50
**#16**	47	IIIC	High-grade serous carcinoma	NA	24.5 cm×14.5 cm×11.5 cm	Yes	24.39
**#17**	48	IV	High-grade serous carcinoma	>1000	7.6 cm×4.5 cm×8.0 cm,8.9 cm×7.6 cm×9.0 cm	No	2.71
**#18**	34	IV	Clear cell carcinoma	431	21.2 cm×18.6 cm×12.7 cm	Yes	18.04
**#19**	54	IA	Borderline mucinous tumor	69.5	9.6 cm×10.1 cm×12.4 cm	No	46.12
**#20**	36	IV	Endometrioid adenocarcinoma	>1000	8.2 cm×4.7 cm×5.6 cm	Yes	14.81
**#21**	47	IIIB	High-grade serous carcinoma	5000	7.1 cm×6.3 cm×5.7 cm	Yes	97.86
**#22**	43	IIIC	High-grade serous carcinoma	>1000	4.8 cm×5.7 cm×3.2 cm,3.1 cm×3.7 cm×2.8 cm	Yes	18.18
**#23**	65	IIIC	Poorly differentiated neuroendocrine carcinoma	>1000	NA	Yes	50.25
**#24**	41	IV	High-grade serous carcinoma	>1000	10.0 cm×8.6 cm×6.3 cm,7.2 cm×6.8 cm×6.5 cm	Yes	339.63
**#25**	59	IIIC	High-grade serous carcinoma	>1000	3.9 cm×2.7 cm,3.5 cm×4.4 cm×2.0 cm	Yes	179.95
**#26**	11	IIIC	Yolk sac tumor	410.6	14 cm×11 cm×13 cm	Yes	102.15
**#27**	61	IIIC	High-grade serous carcinoma	>5100	3 cm×3 cm×2 cm	Yes	23.09

*FIGO* International Federation of Gynecology and Obstetrics, *NA* not available.

**Table 2:** The clinicopathological features of the two ovarian cancer patients for primary tumor cell culture.

No	Age (years)	FIGO stage	Histological type	Neoadjuvant chemotherapy	Tumor size	Chemotherapy
T1	58	IIIC	High-grade serous carcinoma	NO	12.0 cm×10.0 cm×6.5 cm	TP
T2	68	IIIC	High-grade serous carcinoma	NO	5.6 cm×5.6 cm×5.2 cm	TP

*FIGO* International Federation of Gynecology and Obstetrics, *TP*, chemotherapy based on paclitaxel and platinum.

### Cell culture and processing

The human ovarian cancer cell lines A2780, SKOV3, CAOV3, and ES2 were purchased from the cell bank of the Chinese Academy of Sciences and cultured in DMEM/F12 medium supplemented with 10% FBS (Gibco**)**. A2780 is a human ovarian cancer cell line, CAOV3 is a human papillary ovarian adenocarcinoma cell line, while ES2 is a human clear ovarian cell line and SKOV3 is a human ovarian adenocarcinoma cell line. All cell lines were cultured in a humidified incubator at 37 °C in 5% CO_2_ and were authenticated by short tandem repeat (STR) profiling and confirmed to be mycoplasma negative. For exosome processing, ovarian cancer cells were grown to a density of approximately 70% and washed with phosphate buffer saline (PBS) twice to exclude the interference of abundant exosomes in serum. The cancer cells were then cultured with exosome-free serum, and 0 µg/ml, 50 µg/ml, and 100 µg/ml ADEs were added to the culture medium. After 48 h of culture, the cancer cells were collected for subsequent experiments.

### Isolation of ADEs

For the separation of ascites exosomes, we used the classic ultracentrifugation method. The ascites was first centrifuged at 300 × *g* for 10 min, and cell pellets were collected and stored at −80 °C for subsequent immunohistochemistry analysis about cell components. The supernatant was further centrifuged at 2000 × *g* for 20 min, filtered through 0.22-μm filters (Millipore), centrifuged twice with an Optima MAX-XP centrifuge (Beckman Coulter) at 100,000 × *g* for 70 min, and dissolved in PBS. Exosomes were used or stored at −80 °C. Considering that HGSOC is the most common pathology type in ovarian cancer and accounts for 70–80% of ovarian cancer deaths^3^, ADEs #2, #3, #6, #7, #12, #21, #25, and #27, which derived from HGSOC patients, were chosen to conduct most of the experiments. #7 ADEs were used throughout the study because there was a large amount of ascites available, which provided us with abundant exosomes for the experiments. The protein concentration of exosomes was determined by a bicinchoninic acid (BCA) protein kit (Beyotime).

### Transmission electron microscopy (TEM)

Exosomes were observed by TEM using negative staining with copper mesh as in a previous study^23^. Exosomes were dissolved in 10 μl of PBS and then dripped on a copper mesh with a diameter of 2 nm. The sample was air dried for several minutes at room temperature, exposed to 20 μl of phosphotungstic acid solution at a concentration of 3% for several minutes, and then dried at room temperature. The shape and size of the exosomes were finally observed with a MegaView G2 transmission electron microscope.

### Dynamic light scattering (DLS) and NanoSight analysis (NTA)

DLS analysis and NTA analysis were performed in a Zeta Potential Analyzer instrument (Colloidal Dynamics, USA) and a NanoSight NS300 instrument (Malvern, UK). The samples were added to the instrument to analyze the diameter and concertation of exosomes.

### Flow cytometry

Flow cytometric analysis of the surface marker of exosomes was conducted as described previously^24^. Three microliters of latex microspheres (aldehyde/sulfate latex beads, Invitrogen) were added to exosomes with 40 μg of protein and gently mixed for 2–3 h at room temperature. Then, 1 M glycine was added and mixed upside down for 0.5 h to block the reaction, and the sample was centrifuged at 300 × *g* for 10 min. Then, PBS was added and centrifuged to wash away unbound antibody. Primary antibodies against CD63 and CD9 were added and mixed at 4 °C overnight, while rabbit IgG was used as an isotype control. The samples were washed with PBS twice, reacted with a fluorophore-conjugated secondary antibody at room temperature for 1 h, and detected by flow cytometry.

### Exosome uptake assay

For exosome uptake experiments, freshly isolated exosomes were stained with a PKH67 Green Fluorescent Cell Linker Mini Kit (Sigma), and the procedure was conducted according to the instructions. PKH67-pretreated exosomes were added to the cells in culture, and culture was continued for 24 h in the dark. Recipient cells were incubated with phallotoxins (Invitrogen Life Technologies) and reacted for 60 min at room temperature. Finally, the cells were imaged under a fluorescence microscope.

### Migration and invasion assays

A total of 4×10^4^ or 8×10^4^ cancer cells treated with or without ADEs were plated in 24-well transwell plates with inserts (8 μm in diameter, Corning) with or without Matrigel. The medium in the inserts did not contain FBS, whereas the medium outside of the inserts was supplemented with 10% FBS. After 24 h, the cell inserts were fixed with formaldehyde, stained with 0.1% crystal violet, observed and imaged under a microscope.

### Wound-healing assay

For the wound-healing assay, equal numbers of cells treated with ADEs were plated into six-well plates. The cell monolayers were wounded with a pipette tip to create a gap on the plates. After washing the cells gently with PBS to remove floating cells, the culture medium was replaced with serum-free medium to inhibit cell proliferation, and cancer cells were allowed to close the wound for 24 h. The area of wound closure after the first scratch (0 h) and after 24 h (24 h) was observed and measured by microscopy with Image-Pro Plus 6 software (Media Cyberbetics, USA).

### F-actin immunofluorescence

The F-actin immunofluorescence analysis of ovarian cancer cells was conducted as described previously^25^. The cells were fixed with 4% formaldehyde and washed with PBS three times for 5 min each. Then, phallotoxins (Invitrogen Life Technologies) were added and reacted for 60 min. Subsequently, the cell nuclei were stained with DAPI. The cell morphology was imaged under a fluorescence microscope.

### Western blotting

The protein concentration of exosomes was directly measured with BCA assay. Total cellular protein was extracted with radioimmunoprecipitation assay (RIPA) buffer including a protease inhibitor cocktail for 30 min and centrifuged at 13 000 × g for 10 min to collect supernatants, and the protein concentration was measured with BCA assays. Samples were separated by 10% sodium dodecyl sulfate-polyacrylamide gel electrophoresis (SDS-PAGE) and transferred onto polyvinylidene difluoride (PVDF) membranes. Membranes were then blocked with 5% nonfat milk in Tris-buffered saline with Tween 20 (TBST) for 1 h at room temperature and incubated with primary antibodies specific for the following proteins at 4 °C overnight: CD63 (1:1000 dilution, Abcam, ab134045, USA); CD9 (1:1000 dilution, Abcam, ab92716, USA); calnexin (1:2000 dilution, Abcam, ab133615, USA); E-cadherin (1:3000 dilution, CST, #3195 S, USA); N-cadherin (1:1000 dilution, CST, #13116 S, USA); Vimentin (1:1000 dilution, CST, #5741 S, USA); PAX8 (1:5000 dilution, Proteintech, 10336-1-AP, USA); cytokeratin 8 (1:500 dilution, Abcam, ab53280, USA); calretinin (1:1000 dilution, Abcam, ab92341, USA); α-SMA (1:5000 dilution, Abcam, ab5694, USA); and β-actin (1:5000 dilution, Proteintech, 66009-1-Ig, USA). Chemiluminescence detection of protein bands was performed using horseradish peroxidase-conjugated anti-rabbit or anti-mouse secondary antibodies, and the protein bands were detected with an enhanced chemiluminescence kit (Pierce) in a Molecular Imager® ChemiDocTM XRS + system with Image Lab^TM^ software (Bio-Rad).

### Immunofluorescence

The experimental immunofluorescence procedure was the same as that previously described^26^. Slides with cells were washed three times and fixed with 4% paraformaldehyde. After three washes with PBS, the slides were reacted with 0.1% Triton X for 30 min to disrupt the cell membrane and 1% BSA for 30 min to block the antigen at room temperature and were then incubated with the primary antibodies at 4 °C overnight. Then, the slides were washed with PBS on a shaking bed at room temperature for 5 min; this step was repeated 3 times. The slides were then incubated with fluorescence-labeled secondary antibodies for 1 h at room temperature. DAPI was added and incubated for 30 min at room temperature to stain nuclei. After three washes, the cells were imaged under a fluorescence microscope. For membrane proteins, that is, E-cadherin and N-cadherin, the cells were fixed, and the primary antibody was added without disrupting the membrane. After the primary antibody was washed off on the second day, 0.1% Triton X-100 was added, and the following steps were the same as those for cytoplasmic proteins.

### 5-ethynyl-2′- deoxyuridine (EdU) cell proliferation assay

Cancer cells treated with ADEs were digested with trypsin and inoculated in 96-well plates. The EdU cell proliferation assay was conducted according to the instructions of the EdU kit (RiboBio, China). The results were observed and imaged with a fluorescence microscope. The experiment was repeated three times.

### Colony formation assay

Cancer cells treated with ADEs were digested with trypsin and inoculated in 6-well plates at 1000 cells per well. After 7-10 days of culture, the cells grew into visible colonies. The plates were gently washed two times with PBS, fixed for 20 min with 4% polyformaldehyde at room temperature, washed two times with PBS, and stained for 30 min with 0.1% crystal violet at room temperature. The experiment was repeated three times.

### RNA extraction and qRT-PCR

Total RNA was extracted from cells using TRIzol reagent (TaKaRa, Japan) according to the manufacturer’s instructions, and purification of miRNAs from exosomes and qRT-PCR were performed as described previously^27^. Reverse transcription was performed using a miRNA reverse transcription kit (Toyobo, Japan) and random primers. Real-time fluorescence quantitative PCR with SYBR Green was carried out in an Applied Biosystems Plus PCR System (ABI). All miRNA primers were designed and synthesized by RiboBio (China). The expression of intracellular miRNA was normalized to that of U6 as the internal reference gene, while the expression of exosomal miRNA was normalized to that of miR-16 as the internal reference miRNA^28^. Each reaction was performed in triplicate, and the experiment was repeated three times.

### Orthotopic xenograft mouse model of ovarian cancer

All mice used to establish the orthotopic xenograft mouse model of ovarian cancer were housed in the experimental animal center of Tongji Medical College, Huazhong University of Science and Technology. All procedures followed the operational specifications of the animal management committee. Four- to six-week-old female BALB/c nude mice were purchased from Beijing Hua Fukang Biological Polytron Technologies, Inc. The ovarian cancer cell line SKOV3 was transduced with a lentiviral luciferase reporter gene, screened with puromycin, and selected for monoclonal cells, which were termed SKOV3-luc+ cells. SKOV3-luc+ cells in logarithmic growth phase were digested with trypsin, suspended in serum-free medium, and used at a density of 5×10^8^ cells/ml. Nude mice were completely anesthetized (4% chloral hydrate, 0.1 ml/10 g body weight) and placed in the lateral position. An incision in the peritoneum was made in the left abdomen. The incision was made parallel to the spinal column, and the length was 1 cm. After the left ovary was exposed, 5 μl of cell suspension was injected gently, slowly, and meticulously into the ovary to avoid leakage and capsule rupture. The ovarian cavity was returned to the abdominal cavity, and the peritoneum and skin were sutured layer by layer. Approximately 30 min after the operation, the mouse was revived, and observation was continued.

### Effect of ADEs on ovarian cancer in vivo

The orthotopic xenograft mouse model and bioluminescence live imaging were used to detect the effect of ADEs on ovarian cancer in vivo. Twelve nude mice were randomly divided into two groups with 6 mice in each group. The first day after inoculation of SKOV3-luc+ cells into the nude mice, one group received 50 µg of ADEs by intraperitoneal injection every three days, while the control group received PBS by intraperitoneal injection. The first bioluminescence live imaging was performed 7 days after inoculation. The nude mice were anaesthetized with 2% isoflurane and 1.5 L/min oxygen and injected intraperitoneally with D-luciferin at a dose of 150 mg/kg. Ten minutes after substrate injection, the nude mice were placed in the imaging room in the prone position. The intensity and location of luminescence were detected with a live imaging biological instrument. Whole-body live imaging was conducted once a week for 8 weeks.

### Immunohistochemistry (IHC)

Immunocytochemical analysis of E-cadherin, N-cadherin, and vimentin in orthotopic tumor tissues of nude mice was conducted as previously described^29^. Paraffin slides were dehydrated three times with TO clarifier for biological tissue slicing and dehydrated through a gradient ethanol series. Antigen retrieval was performed for 20 min in 0.01 M citric acid solution at 95 °C. Then, 3% H_2_O_2_ and normal sheep serum were used to quench endogenous peroxidase activity and block the antigen, respectively. The tissues were incubated with primary anti-E-cadherin, anti-N-cadherin, anti-Vimentin and anti-Ki67 (Abcam, ab15580, USA) antibodies at 4 °C overnight prior to incubation with a biotinylated goat anti-rabbit antibody for 1 h at room temperature. Immunostaining was performed with diaminobenzidine (DAB). Normal rabbit IgG was used as a negative control, and positive expression was defined as brown-yellow granules distributed in the cytoplasm, nucleus or cytomembrane. Ki67 immunoreactivity was assessed according to the average percentage of positively stained cells in five randomly selected fields. The immunoreactivity scores for E-cadherin, N-cadherin and Vimentin were determined by Image-Pro Plus, version 6.0 (Media Cyberbetics, USA) as follows: Mean Density = Integrated Optical Density/Area of DAB staining^30^.

### Survival analysis of miR-6780b-5p in ovarian cancer patients

To determine the prognostic significance of miR-6780b-5p in ovarian cancer patients, we used the online microarray data analysis website K-M Plotter^31^ to investigate the prognostic significance of miR-6780b-5p in pancancer datasets. “miRNA-pancancer analysis” was selected. “miR-6780b” was entered as gene symbol, and the “Auto select best cut off” button was selected. Kaplan–Meier and log-rank test were used to evaluate differences in overall survival (OS) time.

### Statistical analysis

Statistical analysis was performed using SPSS 17.0 statistical software and GraphPad Prism 5.0 software. Numerical data are expressed as the mean ± standard deviation (s.d.) values. Student’s *t* test or one-way analysis of variance was used to evaluate the differences between two or multiple groups, respectively; *p* < 0.05 was considered significant.

## Results

### ADEs enter ovarian cancer cells

**Fig. 1:**
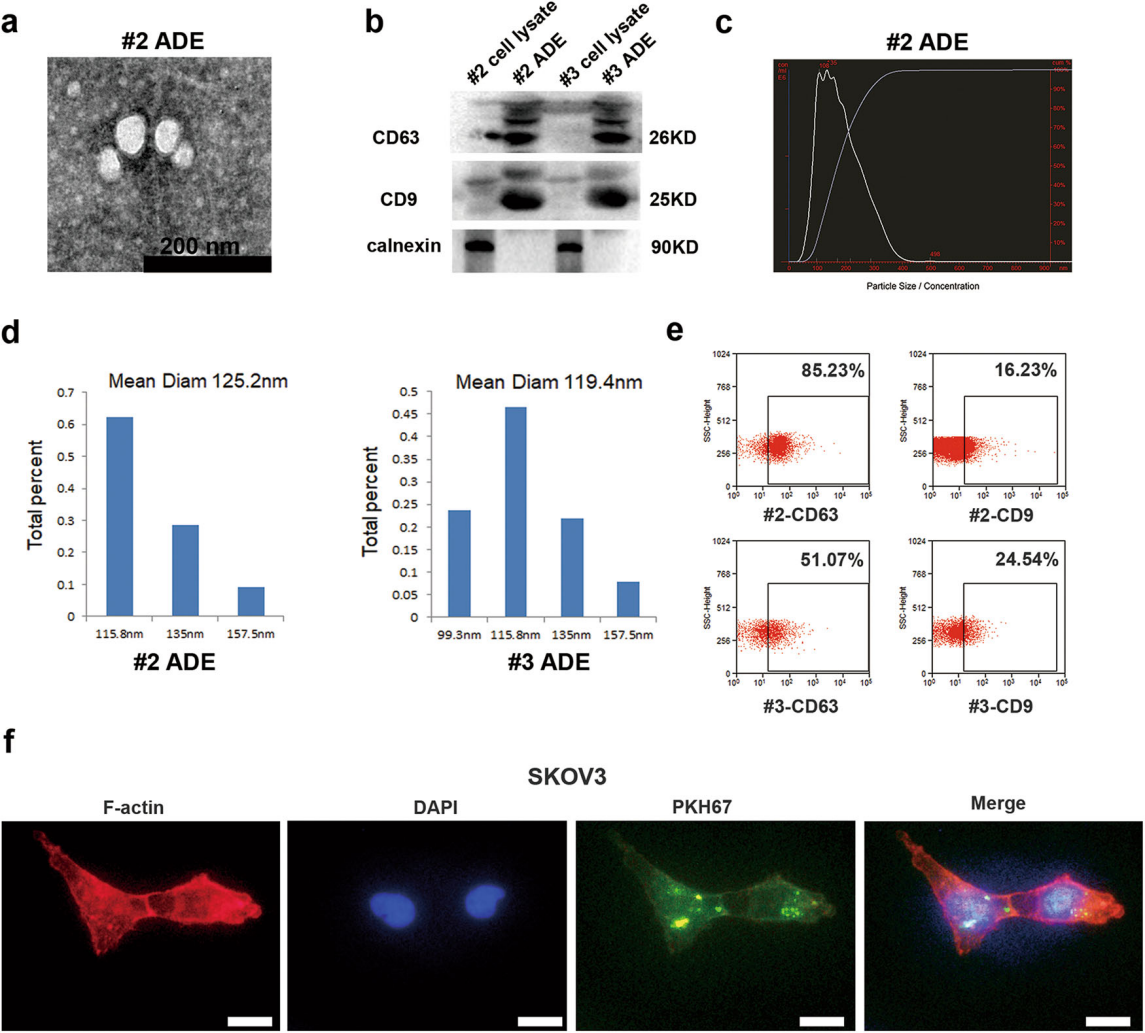
ADEs enter ovarian cancer cells. **a** Electron micrograph of the exosomes extracted from ascites of patient #2. Scale bar, 200 nm. **b** Western blot analysis of CD63, CD9 and calnexin in ADEs #2 and #3 as well as in the cell pellet control. **c** Size analysis of the ADEs #2 and #3 using NTA. **d** Size analysis of ADEs #2 and #3 using DLS analysis. **e** Flow cytometric analysis of the expression of CD63 and CD9 in ADEs #2 and #3. **f** Immunofluorescence detection of ADE uptake by SKOV3 cells. In exosomes, PKH67 was labeled with green, and F-actin was labeled red; nuclei were labeled with DAPI (blue). Scale bar, 20 μm.

The four kinds of ADEs used in the subsequent experiments, which were obtained from four HGSOC patients, including #2, #3, #7 and #27, were characterized and quantified by TEM, Western blot analysis, DLS, NTA and flow cytometry. And the cell pellets from #7 and #27 patients were detected by immunohistochemistry to show the cell components in the ascites. ADEs appeared as round vesicles with a double layer, and a diameter of 50-140 nm under an electron microscope (Fig. 1a, Supplementary Fig. 1a), consistent with the NTA and DLS analysis results (Fig. 1c, d, Supplementary Fig. 1c, d). Western blot analysis showed that CD63 and CD9, markers of exosomes, were highly expressed in ADEs compared with cell pellets, while calnexin, a negative control for exosomes^32,33^, was not present in ADEs but existed in cell pellets (Fig. 1b, Supplementary Fig. 1b). Furthermore, we used flow cytometry to analyze the expression of surface marker proteins of ADEs. We found that ADEs from different patients displayed diverse expression of surface marker proteins. For example, ADEs #2 and #3 expressed high levels of CD63, with positive rates of 85.23% and 51.07%; in addition, the positive rates of CD9 expression were 16.23% and 24.54%, respectively (Fig. 1e). To confirm whether ADEs can enter ovarian cancer cells, we used PKH67 to label #7 ADEs and conducted immunofluorescence to track the location of ADEs in ovarian cancer cells. The results showed that green fluorescently labeled ADEs could enter SKOV3, A2780, CAOV3 and ES2 cells (Fig. 1f, Supplementary Fig. 2). In addition, IHC analysis of cell pellets from #7 and #27 ascites were conducted to explore the origin cells of ADEs. It showed that various cell types existed in the ascites, including cancer cells, CAFs, and macrophagocytes (Supplementary Fig. 3), implying ADEs may be secreted by those cells.

### ADEs promote EMT of ovarian cancer cells in vitro

**Fig. 2:**
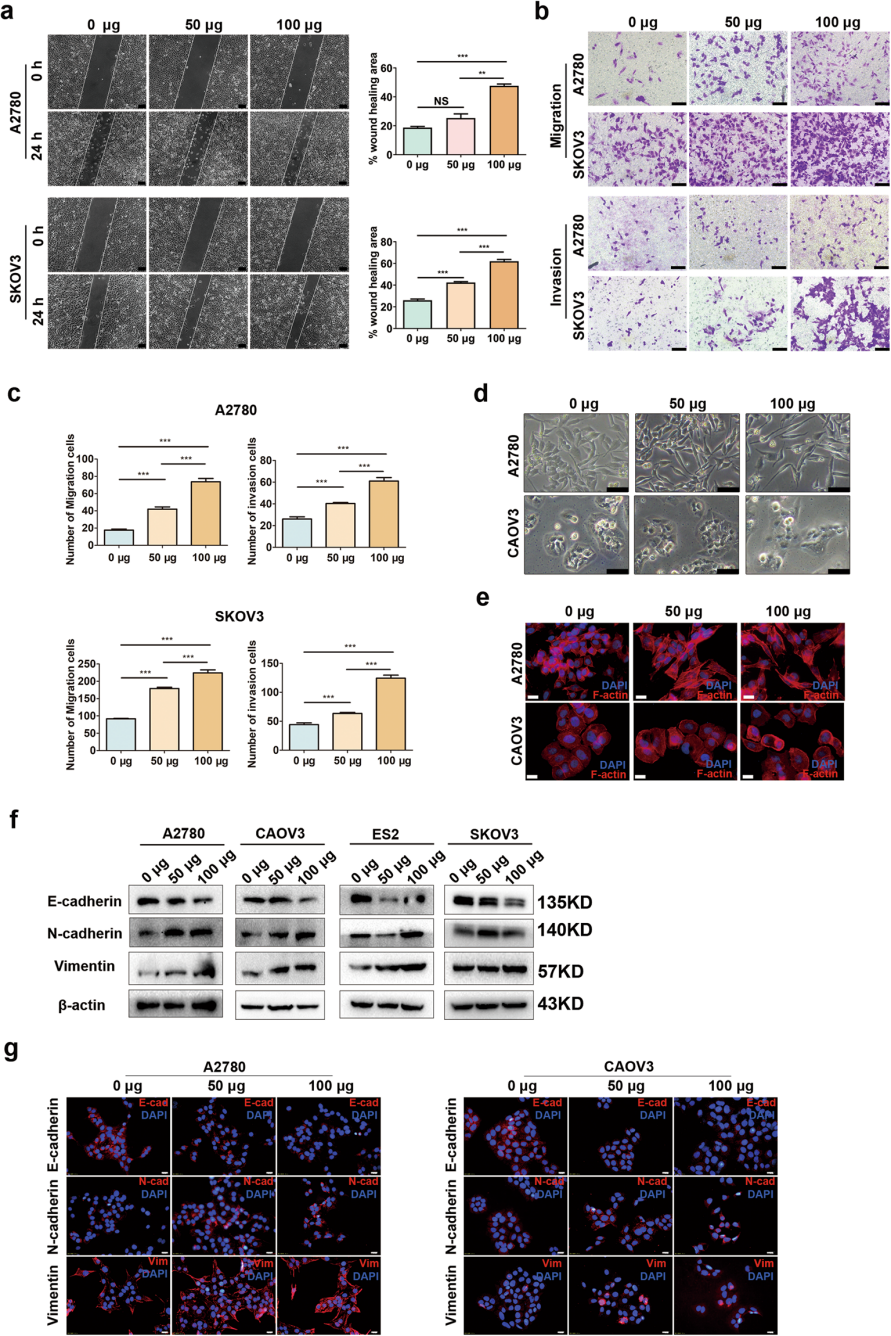
ADEs promote EMT of ovarian cancer cells in vitro. A2780, CAOV3, ES2 and SKOV3 ovarian cancer cells were treated with 0 μg, 50 μg, and 100 μg of #7 ADEs. **a** Representative images of wound-healing assays of A2780 and SKOV3 and the area of wound closure was measured. Scale bar, 100 μm. **b** Representative images from migration and invasion assays of A2780 and SKOV3 cells. Scale bar, 100 μm. **c** Invaded and migrated cells were counted and are shown as histograms. **d**, **e** Changes in the morphology of A2780 and CAOV3 cells were evaluated by microscopic observation and F-actin staining. Scale bar, 100 μm (**d**), Scale bar, 20 μm (**e**). **f** Western blot analysis of E-cadherin, N-cadherin and Vimentin expressions in A2780, CAOV3, ES2 and SKOV3 cells. **g** Immunofluorescence analysis of E-cadherin, N-cadherin, and Vimentin in A2780 and CAOV3 cells. Experiments were performed in at least triplicate, and the results are shown as the mean ± s.d. values. Student’s *t* test was used to analyze the data (NS, not significant; *p < 0.05; **p < 0.01; ***p < 0.001). Scale bar, 20 μm.

To clarify the effects of ADEs on the invasion and migration of ovarian cancer cells, #7 ADEs extracted from a HGSOC patient were chosen to explore the effects of ADEs on three ovarian cancer cell lines, that is, A2780 and CAOV3 cells, which had reduced tendency to metastasize, and SKOV3 cells, which was more capable of metastasis. Wound-healing assays and transwell migration and invasion assays showed that ADEs significantly promoted the migration and invasion of each cell line in vitro in a concentration-dependent manner (Fig. 2a–c), while CAOV3 cells were absent because their invasion and migration abilities were too low to pass through the transwell membranes or moved closer. We also found that A2780 and CAOV3, which seemed to be more similar to epithelial phenotype and had relatively reduced tendency to metastasize, acquired an elongated, dispersed, and more spindle-shaped morphology after treatment with the ADEs (Fig. 2d, e), indicating the probable role of ADEs in EMT. The Western blot and immunofluorescence results showed that the #7 ADEs facilitated EMT of ovarian cancer cells (Fig. 2f, g), consistent with the results for #27 ADEs (Supplementary Fig. 4). In addition, the EdU cell proliferation and colony formation assays indicated that ADEs accelerated the proliferation of ovarian cancer cells (Supplementary Fig. 5).

### ADEs promote EMT and metastasis of ovarian cancer cells in vivo

**Fig. 3:**
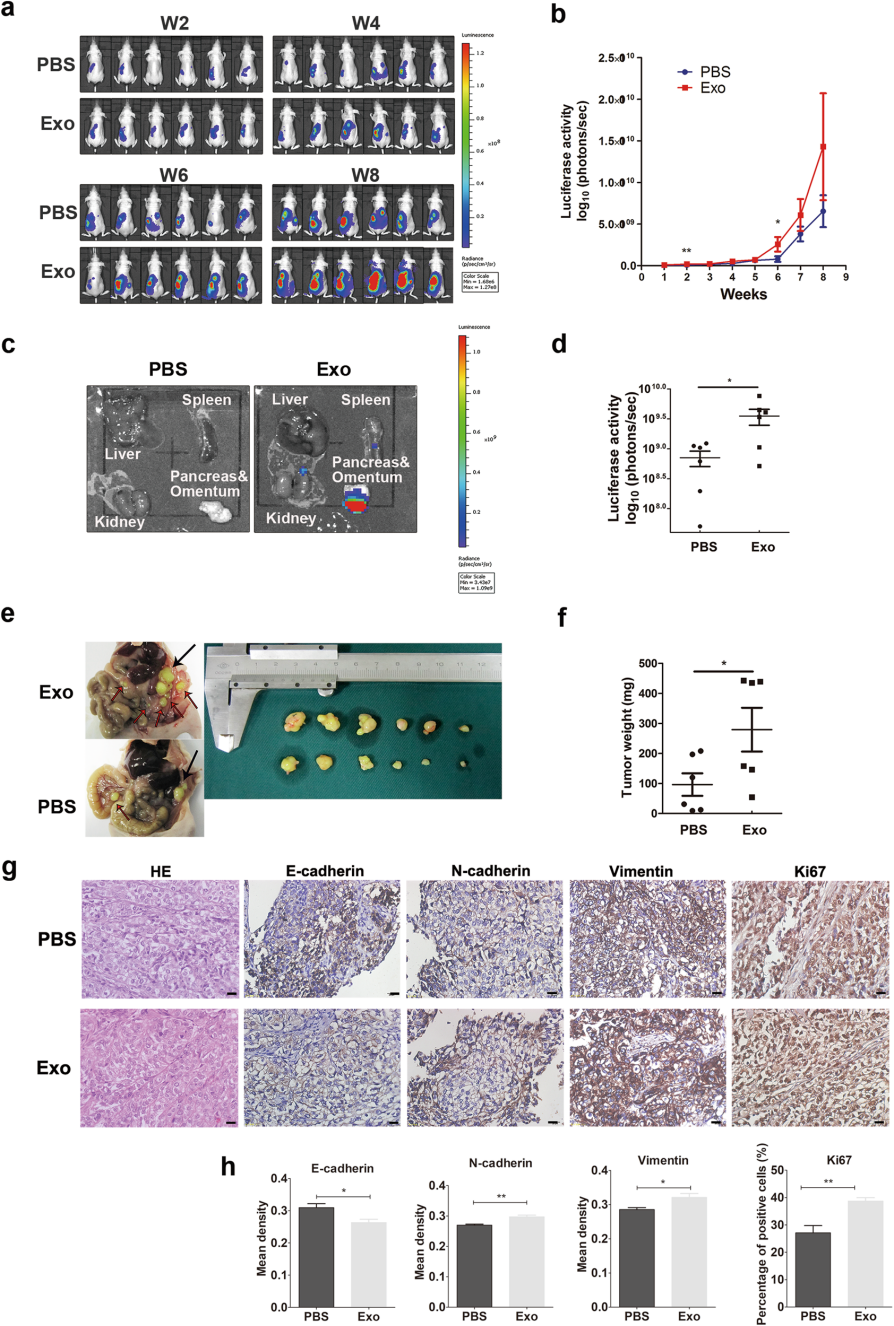
ADEs promote metastasis and EMT of ovarian cancer in vivo. Mice bearing SKOV3-luc orthotopic xenotransplants were injected intraperitoneally with #7 ADEs or PBS. **a** Bioluminescence images of abdominal metastasis in mice bearing SKOV3-luc orthotopic xenotransplants. **b** Growth curve of SKOV3-luc tumors based on the quantitative radiance values from IVIS imaging. (**c**) Bioluminescence images of metastases on abdominal organs. The organs arranged clockwise from the top are the liver, spleen, kidneys, pancreas, and omentum. **d** Quantitative radiance values from IVIS imaging in metastases on abdominal organs. **e**, **f** The size and weight of the ovarian primary tumors from each group of nude mice. **g** Immunohistochemical analysis of the expression of E-cadherin, N-cadherin, and Vimentin in tumor tissues. Scale bar, 20 μm. **h** Diagram of the calculated percentage of Ki67 staining and mean density of E-cadherin, N-cadherin, and Vimentin in the two groups (*p < 0.05; **p < 0.01; ***p < 0.001).

To further explore the effects of ADEs on the metastasis of ovarian cancer in vivo, we established an orthotopic xenograft model of ovarian cancer in nude mice. In vivo imaging technology was used to continuously observe the growth and metastasis of ovarian cancer. Two sets of ADEs from two HGSOC patients, i.e., ADEs #7 and #27, were intraperitoneally injected every three days. The results showed that intraperitoneal injection of #7 ADEs accelerated tumor growth (Fig. 3a, b) and metastasis to the abdominal organs (Fig. 3c, d). Mice in the ADE-treated group had larger tumors and more peritoneal metastases than those in the control group (Fig. 3e, f). These results demonstrated that ADEs promote ovarian cancer metastasis. We also conducted immunohistochemical analysis of E-cadherin, N-cadherin, and Vimentin in tumor tissues of orthotopic xenograft model mice and verified that ADE treatment decreased the expression of E-cadherin and increased the expression of N-cadherin and Vimentin in the orthotopic tumors (Fig. 3g, e), indicating the transition of cells from an epithelial to a mesenchymal phenotype in the tumor tissue. The same effect was observed with the #27 ADEs (Supplemental Figure 6).

### ADEs promote EMT of primary tumor cells

**Fig. 4:**
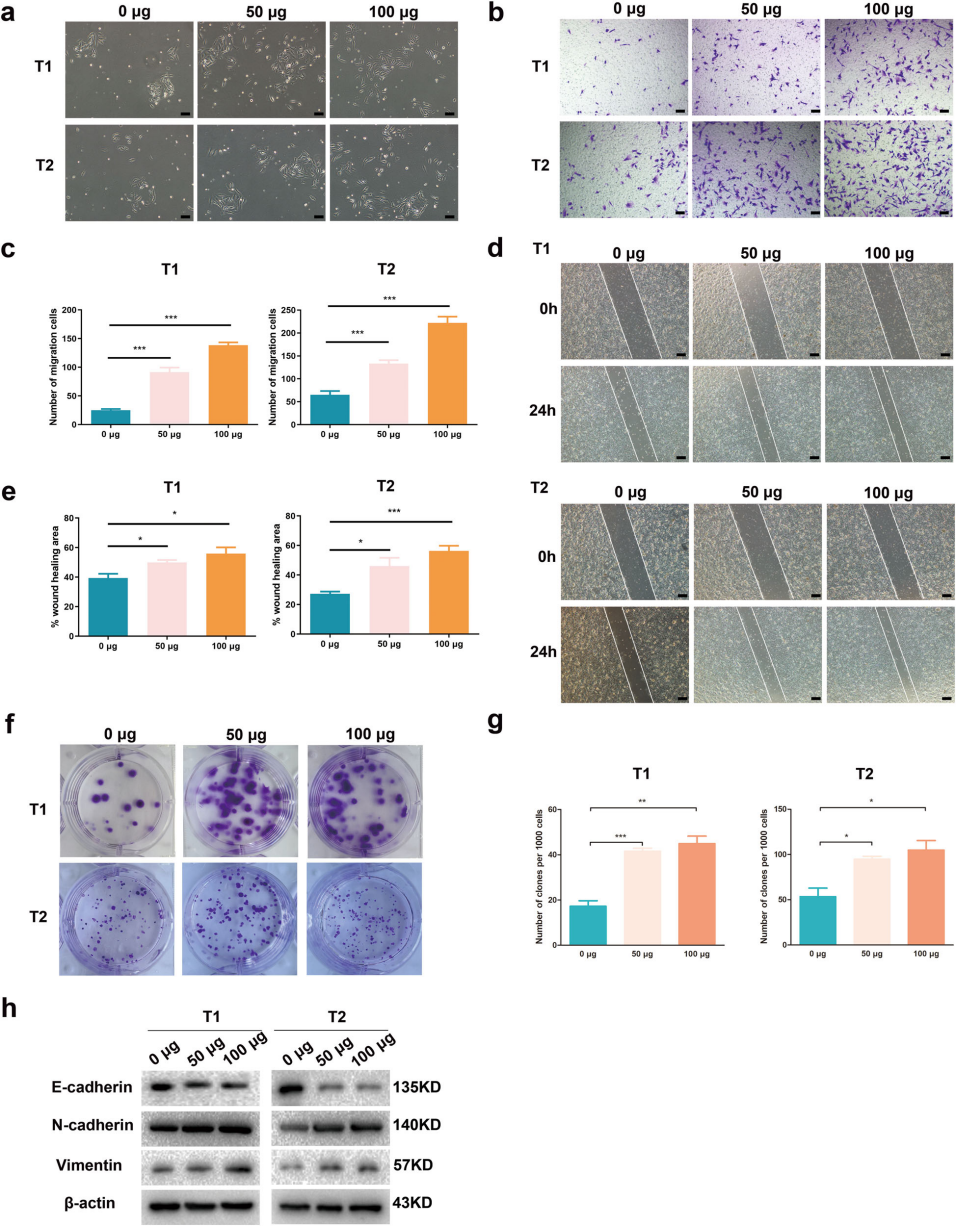
ADEs promote EMT of primary tumor cells. Primary tumor cells extracted from two patients were treated with 0 μg, 50 μg, or 100 μg of #7 ADEs. **a** Representative images of morphological changes in primary tumor cells. Scale bar, 100 μm. **b** Representative images of in vitro transwell migration assays of primary tumor cells. Scale bar, 100 μm. **c** The numbers of migrated cells in transwell migration assays were counted and are shown as histograms. **d** Representative images of in vitro wound-healing assays of primary tumor cells. Scale bar, 200 μm. **e** The areas of wound closure were measured and are shown as histograms. **f** Representative images of in vitro colony formation assays of primary tumor cells. **g** The percentages of cells forming colonies are shown as histograms. **h** Western blot analysis of the changes in the expression of EMT markers in primary tumor cells. Experiments were performed in at least triplicate, and the results are shown as the mean ± s.d. values. Student’s *t* test was used to analyze the data (NS, not significant; *p < 0.05; **p < 0.01; ***p < 0.001).

We confirmed that ADEs promote ovarian cancer cell invasion and metastasis in vitro and in vivo by facilitating EMT in cancer cells. To further clarify the effect of ADEs on primary tumor cells extracted from patient’s tissue, we detected the impact of ADEs on cell migration and proliferation. Surgically removed cancer tissues from two HGSOC patients was used to culture primary tumor cells, which showed a characteristic cobblestone appearance before treatment with ADEs (Supplementary Fig. 7a). Western blot analysis, flow cytometry and immunohistochemical staining for specific markers confirmed the epithelial nature of the primary cells as well as the absence of other contaminating cell types (Supplementary Fig. 7b–d). After treatment with #7 ADEs, the primary tumor cells (T1 and T2) became slender and dispersed (Fig. 4a), and the migration as well as proliferation of the primary tumor cells was accelerated by ADE treatment (Fig. 4b–g). ADE treatment decreased the expression of E-cadherin and increased the expression of N-cadherin and Vimentin in the primary tumor cells (Fig. 4h).

### ADEs transfer miR-6780b-5p to ovarian cancer cells

**Fig. 5:**
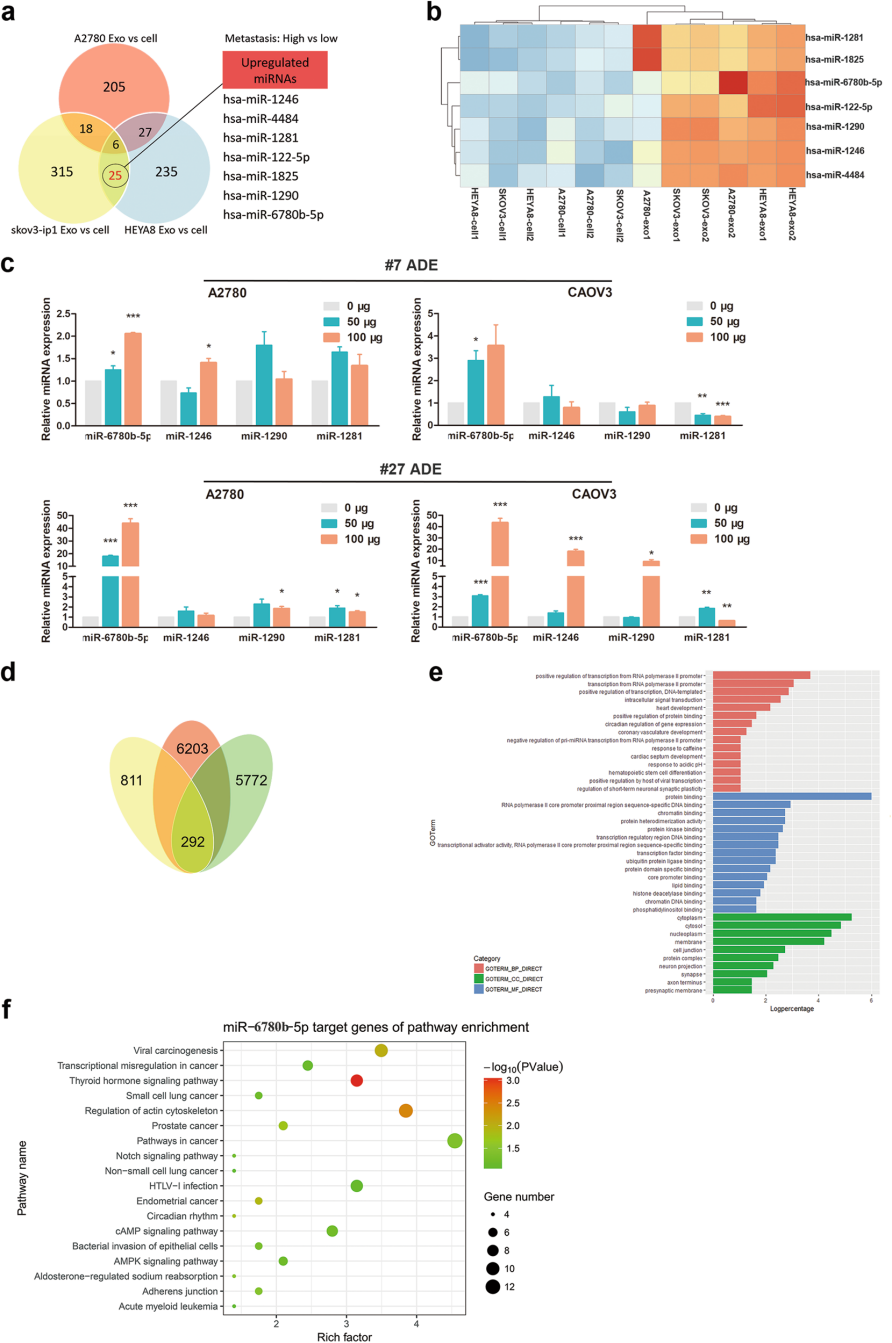
ADEs transfer miR-6780b-5p to ovarian cancer cells. **a** The expression of miRNA in the exosomes from three ovarian cancer cell lines was compared with that in the cytoplasm, and samples with higher expression were selected. A total of 25 miRNAs were expressed in aggressive SKOV3-ip1 and HEYA8 cells but not in the less-invasive A2780 cells. **b** Thermograms of the seven miRNAs in ovarian cancer cells with the highest expression among the 25 miRNAs. **c** qRT-PCR was performed to detect the expression of the seven miRNAs in A2780 and CAOV3 cells treated with #7 or #27 ADEs. (miR-1825, miR-122-5p, and miR-4484 are not shown because their CT values were greater than 30). **d** Venn diagram of the downstream target genes of miR-6780b-5p, which were predicted with three common miRNA prediction websites: TargetScan, miRDB and RNA22. **e** GO (gene function annotation) analysis of the target genes of miR-6780b-5p. **f** KEGG analysis of the possible target genes of miR-6780b-5p.

Data mining based on public databases is currently a popular approach. We searched the ovarian cancer exon chip information in the Gene Expression Omnibus (GEO) public database and downloaded the data set GSE76449^14^, a noncoding RNA profiling array of 6 different ovarian cancer cell lines and one normal ovarian cell line. Bioinformatics analysis of GSE76449 was performed, and 25 miRNAs were found to be more highly expressed in exosomes from the highly metastatic SKOV3-ip1 and HEYA8 cell lines than in exosomes from low-metastatic A2780 cells. Seven candidate miRNAs were the most abundant among the 25 miRNAs (miR-6780b-5p, miR-122-5p, miR-1246, miR-1281, miR-1290, miRNA-4484, and miR-1825) (Fig. 5a, b). To determine which miRNA can be transferred from ADEs to ovarian cancer cells, we used qRT-PCR to detect the expression of the seven miRNAs in A2780 and CAOV3 cells treated with #7 ADEs and #27 ADEs. The results showed that the miR-6780b-5p level increased in a concentration-dependent manner (Fig. 5c), suggesting that miR-6780b-5p may be transferred to ovarian cancer cells by ADEs. Considering that miR-6780b-5p is a new miRNA, we predicted the target genes and functions of miR-6780b-5p with three online miRNA target analysis websites (TargetScan, miRDB and RNA22) (Fig. 5d). Gene Ontology (GO) analysis of miR-6780b-5p target genes showed that miR-6780b-5p may play important roles in many pathophysiological processes, including intercellular signal transduction, transcriptional regulation, and protein binding (Fig. 5e). Moreover, Kyoto Encyclopedia of Genes and Genomes (KEGG) analysis of these target genes was performed with the Database for Annotation, Visualization and Integrated Discovery (DAVID) online website and showed that miR-6780b-5p was related mainly to cytoskeletal regulation, the Notch pathway, and the Mitogen-Activated Protein Kinase (MAPK) pathway (Fig. 5f).

### miR-6780b-5p promotes EMT in ovarian cancer cells

**Fig. 6:**
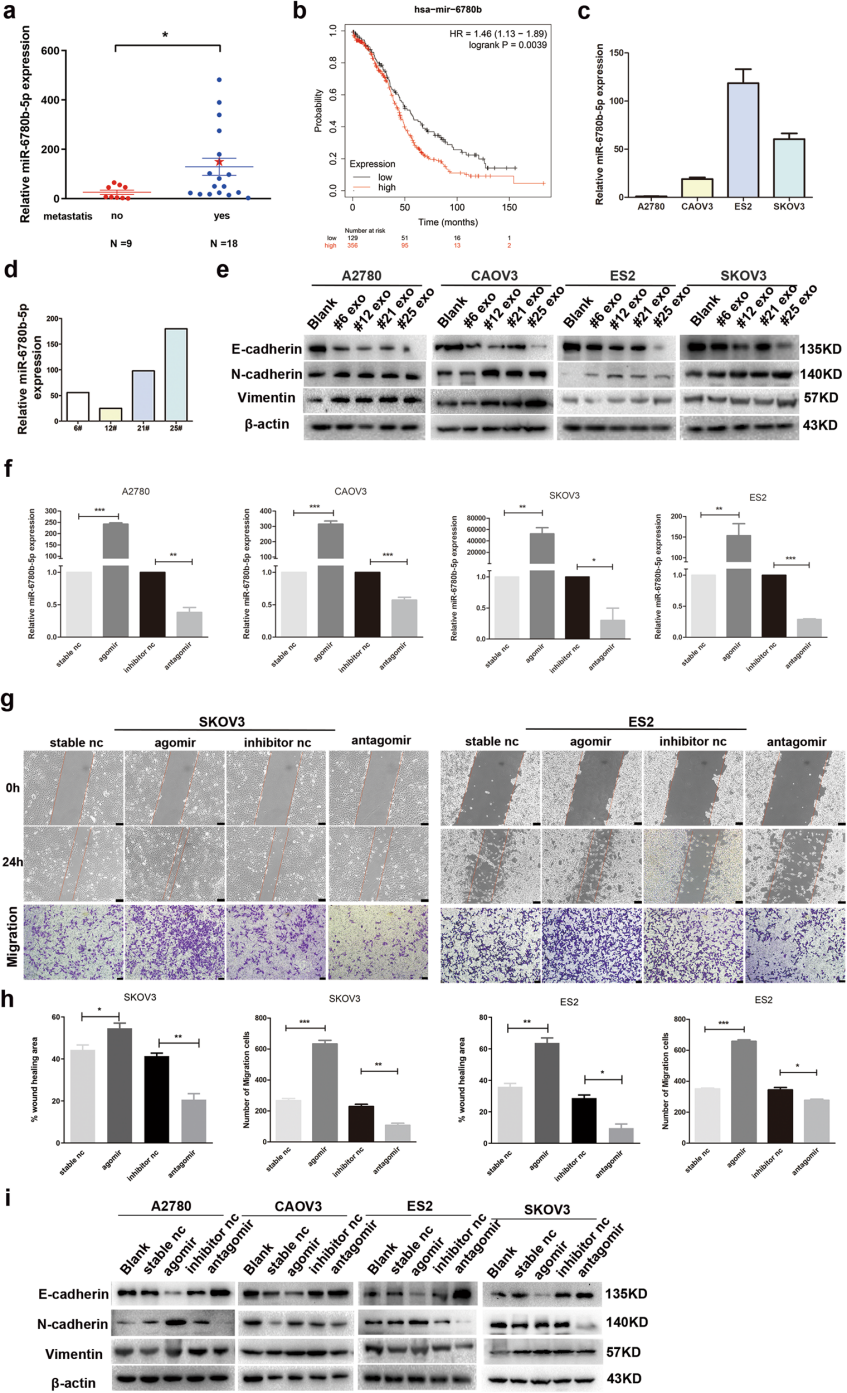
miR-6780b-5p promotes EMT in ovarian cancer cells. **a** qRT-PCR was used to compare miR-6780b-5p expression among 18 ADEs with peritoneal metastasis and nine without peritoneal metastasis. ★, #7 ADEs. **b** Kaplan–Meier analysis of miR-6780b in pancancer analysis with K-M Plotter. **c** qRT-PCR was used to compare miR-6780b-5p expression in four ovarian cancer cell lines. **d** qRT-PCR was used to compare the relative miR-6780b-5p expression in four sets of ADEs (#6, #12, #21, #25) from four HGSOC patients. **e** Western blots of E-cadherin, N-cadherin and Vimentin expression in A2780, CAOV3, ES2, and SKOV3 cells treated with four sets of ADEs with different miR-6780b-5p concentrations. **f** qPCR validation of the effects of the miR-6780b-5p agomir and antagomir in A2780, CAOV3, ES2, and SKOV3 cells. **g** Representative images of in vitro wound-healing and transwell migration assays with SKOV3 and ES2 ovarian cancer cells after transfection of miR-6780b-5p stable NC/agomir and inhibitor NC/antagomir. Scale bar for the wound-healing assay, 100 μm. Scale bar for the transwell assay, 100 μm. **h** The areas of wound closure and the numbers of migrating cells in transwell migration assays were measured and are shown as histograms. **i** Western blot analysis of the expression of E-cadherin, N-cadherin, and Vimentin in A2780, CAOV3, ES2, and SKOV3 cells after transfection with miR-6780b-5p agomir and antagomir. Experiments were performed in at least triplicate, and the results are shown as the mean ± s.d. values. Student’s *t* test was used to analyze the data (NS, not significant; *p < 0.05; **p < 0.01; ***p < 0.001).

To clarify the effect of miR-6780b-5p on ovarian cancer, we detected miR-6780b-5p expression in exosomes from 27 ovarian cancer patients. We found that miR-6780b-5p expression was higher in patients who had peritoneal metastasis than those who did not (Fig. 6a). Among the 27 ADEs, 7# ADEs had relatively high expression of miR-6780b-5p (Table 1 and Fig. 6a). In the pancancer miRNA analysis conducted with K-M Plotter^34^, high miR-6780b expression indicated a poor patient survival (Fig. 6b), though the online analysis tool did not distinguish between −3p and the −5p miRNA. The result supports the hypothesis that miR-6780b-5p relates to poor patient survival to some extent. Moreover, we found that miR-6780b-5p expression was higher in SKOV3 and ES2 cells than in A2780 and CAOV3 cells, which have comparatively low migration and invasion abilities (Fig. 6c). To further investigate whether miR-6780b-5p affects EMT of ovarian cancer cells, four sets of ADEs from four HGSOC patients, ADEs #6, #12, #21, and #27, with different miR-6780b-5p concentrations, were used (Fig. 6d). Western blotting revealed that the E-cadherin level in A2780, CAOV3, ES2, and SKOV3 cells was decreased to varying degrees, while the N-cadherin and Vimentin levels were increased, in a miR-6780b-5p level-dependent manner (Fig. 6e). For instance, #25 ADEs had the highest level of miR-6780b-5p, and the corresponding changes in E-cadherin, N-cadherin, and Vimentin levels in ovarian cancer cells after treatment were substantially larger than the changes after treatment with #6, #12, or #21 ADEs, which had relatively low levels of miR-6780b-5p. To further verify the function of miR-6780b-5p in EMT, we used a miRNA agomir and antagomir to alter miR-6780b-5p expression in ovarian cancer cells. The regulatory efficiency was confirmed by qRT-PCR (Fig. 6f). We found that the cancer cells acquired an elongated, dispersed, and more spindle-shaped morphology after treatment with the miR-6780b-5p agomir (Supplementary Fig. 8); moreover, upregulation of miR-6780b-5p promoted and downregulation of miR-6780b-5p suppressed the migration of ES2 and SKOV3 cells (Fig. 6g, h). Western blot analysis showed that upregulation of miR-6780b-5p decreased the expression of E-cadherin and increased that of N-cadherin and Vimentin and that downregulation of miR-6780b-5p had the opposite effects (Fig. 6i). These results suggested that miR-6780b-5p promotes EMT of ovarian cancer cells.

## Discussion

Intraperitoneal spread of a malignancy is orchestrated by a reciprocal interplay between various cells in the tumor microenvironment, including cancer-associated fibroblasts (CAFs), tumor-associated macrophages (TAMs), peritoneal mesothelial cells (PMCs), etc.^35,36^. Because ascites fluid is one of the major contributors to this interaction in the malignancy of ovarian cancer^7,35^, understanding the signal transduction and interactions between ascites fluid and tumor cells in the tumor microenvironment is extremely important. Consistent with previous studies^11^, a large number of exosomes was found in the ascites of ovarian cancer patients and was shown to contribute to tumor progression in our study. Currently, several studies have focused on a single type of exosome from specific kinds of cells. For example, cancer-associated adipocytes (CAAs) and CAFs stimulate ovarian cancer cell motility and invasion via miR-21 delivery^23^. TAM-derived exosomes transfer miR-29a-3p and miR-21-5p to synergistically induce a Treg/Th17 cell imbalance and drive the progression and metastasis of epithelial ovarian cancer (EOC)^37^. Conversely, exosomes derived from hypoxic EOC cells deliver miRNAs to TAMs and elicit a tumor-promoting phenotype^16^. These findings indicate that cells in the intraperitoneal microenvironment transduce signals and interact with each other. Consistent with previous research^38^, we found that various cell types exist in the ascites population, including cancer cells, CAFs and macrophagocytes (Supplementary Fig. 3). Consequently, the ultimate function of ascites or ADEs on tumor biological behavior may be a superposition of effects because ADEs come from these different cells, while the cancer cells are likely the biggest contributor because they were the main composition of ascitic cells (Supplementary Fig. 3). However, no common exosomal miRNA in exosomes from different cells was identified in the present, which increases the difficulty in using a specific miRNA as a therapeutic target to achieve maximum inhibition of tumor metastasis. In our study, we showed that ADEs promoted ovarian cancer metastasis by transferring miR-6780b-5p, a new miRNA, and that inhibition of miR-6780b-5p significantly reduced ovarian cancer cell migration, indicating that exosomal miR-6780b-5p plays an extremely important role in autocrine and paracrine pathways of tumor metastasis. miR-6780b-5p may be the main and common contributing determinant among the various cells in the tumor microenvironment, having value for designing a therapeutic regimen for ovarian cancer metastasis. However, the effect of ADEs cannot be completely explained by the miR-6780b-5p concentration. That is, miR-6780b-5p expression was lowest in #12 ADEs, but large changes in EMT markers were observed in ovarian cancer cells (Fig. 6e), suggesting that some other factors, such as the origin of the ADEs, may play a role in the promotive function on ovarian cancer in addition to the miR-6780b-5p concentration in ADEs. Considering the existence and variable proportions of different kinds of cells in the intraperitoneal microenvironment, it would be helpful to conduct flow cytometry or immunohistochemical staining of the cell pellets to determine the proportions of different cell types within ascites used for exosome isolation. This issue deserves further study.

Epithelial cells located at the leading edge of cancer nests can break through the barrier by undergoing EMT^39^. Many well-known EMT inducers, including proteins and miRNAs, can be transferred by exosomes^40,41^. For example, Notch-1, MMPs, miR-100, LMP1, and HIF alpha promote EMT and tumor metastasis^40,42–44^. In addition, exosomal proteins can perform cell-independent miRNA biogenesis and promote tumorigenesis^45^, indicating the interaction of different components in cancer exosomes. In ovarian cancer, a few studies have focused on cancer exosomes and EMT of cancer cells. W. Li, X. Zhang, et al revealed that TGFβ1 in fibroblast-derived exosomes promotes EMT in ovarian cancer cells^46^, and another team found that high LIN28A-expressing exosomes from ovarian cancer cells induce EMT-related gene expression when taken up by nonmetastatic target cells^47^. In this study, we found that ADEs significantly promoted EMT and metastasis of ovarian cancer cells by transferring miR-6780b-5p. We predicted the function and target genes of miR-6780b-5p. It has been shown that miR-6780b-5p is related mainly to cytoskeletal regulation, the Notch pathway and the MAPK pathway, which are closely related to EMT and tumor metastasis^48,49^. Consistent with the prediction, we observed that the cancer cells acquired an elongated, dispersed, and more spindle-shaped morphology after treatment with 6780b-5p-rich ADEs (Fig. 2d–e**)** or miR-6780b-5p agomir (Supplementary Fig. 8). Thus, we elucidated a new molecular mechanism for promoting exosomal miRNAs in ovarian cancer. In addition, the protein components in ADE have not yet been analyzed; therefore, the interaction between different elements in ADEs should be further investigated to illuminate the complicated function of ADEs.

We realize the emerging therapeutic value of exosomes in cancer metastasis^10^. The advantages of exosomes are as follows: first, exosomes provide a relatively stable environment for therapeutic components, and they can be modified specifically on their membrane or in their contents; moreover, the immune response to exosome-based therapies is minimized^50^. An increasing number of studies are focusing on cancer therapy by altering exosomal secretion or composition of noncoding RNAs^51^, including miRNAs. Katakowski Mark et al. reported that intratumoral injection of miR-146b-modified exosomes significantly reduced tumor volume^52^, while the delivery of miR-9-altered exosomes by mesenchymal stem cells reversed drug resistance. In ovarian cancer, exosomes are considered prospective immunotherapies and therapeutic targets modifying the tumor microenvironment^53^. To our knowledge, miR-21 and miR-29a in exosomes of ovarian cancer promote cancer metastasis^11^, while miR-6126 inhibits the invasion and metastasis of ovarian cancer^14^. These miRNAs may be future candidates for ovarian cancer therapy. In this study, we used bioinformatics methods to screen the key miRNAs related to metastasis and finally identified miR-6780b-5p as the key promotive miRNA in ADEs. These results indicate that miR-6780b-5p may be an important potential therapeutic target for ovarian cancer metastasis.

In conclusion, ADEs promote ovarian cancer metastasis by transferring miR-6780b-5p to ovarian cancer cells, which leads to EMT of these cells and finally enhances their migration, invasion and proliferation. These findings link exosomes with EMT in ovarian cancer and identify a new key exosomal miRNA that plays important roles in metastasis, providing a new target for ovarian cancer treatment and new insight into the mechanisms underlying metastasis in ovarian cancer.

## Supplementary information

Supplementary Information

Suplemental Figure 1

Suplemental Figure 2

Suplemental Figure 3

Suplemental Figure 4

Suplemental Figure 5

Suplemental Figure 6

Suplemental Figure 7

Suplemental Figure 8
