# Supplementary material for: Exosomes in ovarian cancer ascites promote epithelial–mesenchymal transition of ovarian cancer cells by delivery of miR-6780b-5p
Source: Cell Death Dis. 2021 Feb 24;12(2):210. doi: 10.1038/s41419-021-03490-5 (PMC7904844; doi:10.1038/s41419-021-03490-5)
Supplement: Supplementary file 1 — Supplementary Information [file 41419_2021_3490_MOESM1_ESM.docx]

**Supplemental Information**

**Exosomes in ovarian cancer ascites promote epithelial-mesenchymal transition of ovarian cancer cells by delivery of miR-6780b-5p**

Jing Cai, Lanqing Gong, Guodong Li, Jing Guo, Xiaoqing Yi and Zehua Wang

**Supplemental Information contains:**

**-** Supplemental Figure Legends.

- Supplemental Experimental Procedures.

- Supplemental References.

**Supplemental figure legends**

**Supplemental Figure 1. Characteristics of ADEs #7 and #27.** (a) Electron micrograph of the ADEs #7 and #27. Scale bar, 200 nm. (b) Western blot analysis of CD63, CD9, and calnexin in the ADEs #7 and #27. (c) Size analysis of ADEs using NTA. (d) Size analysis of ADEs using DLS. (e) Flow cytometric analysis of the expression of CD63 and CD81 in ADEs #7 and #27.

**Supplemental Figure 2. ADEs enter ovarian cancer cells.** (a-c) Immunofluorescence detection of ADE uptake by A2780, CAOV3 and ES2 cells. In exosomes, PKH67 was labeled with green, and F-actin was labeled red; nuclei were labeled with DAPI (blue). Scale bar, 20 μm.

**Supplemental Figure 3. Ascites of ovarian cancer patients contain various cell types.** The cell pellets of ascites from #7 and #27 patients were used for IHC analysis. (a) Representative images of immunohistochemistry staining for PAX8, E-cadherin, cytokeratin 8, Vimentin, α-SMA and CD68 in embedded sections of ascitic cells. Scale bar, 20 μm.

**Supplemental Figure 4. ADEs promote EMT in ovarian cancer in vitro.** A2780, CAOV3, ES2 and SKOV3 cells were treated with 0 μg, 50 μg, and 100 μg of #27 ADEs. Western blot analysis was performed to evaluate changes in the expression of EMT markers. Experiments were performed in at least triplicate.

**Supplemental Figure 5. ADEs promote the growth of ovarian cancer cells in vitro.** A2780, SKOV3 and ES2 ovarian cancer cells were treated with 0 μg, 50 μg, or 100 μg of #7 ADEs. (a-c) Representative images of EdU proliferation assays of A2780, SKOV3 and ES2 ovarian cancer cells. Scale bar, 20 μm. (d) The percentages of EdU-positive cells in EdU proliferation assays were determined and are shown as histograms. (e) A colony-formation assay was used to evaluate the proliferation of A2780, SKOV3 and ES2 ovarian cancer cells. (f) The numbers of colonies in the colony formation assay were determined and are shown as histograms. Experiments were performed in at least triplicate, and the results are shown as the mean ± s.d. values. Student’s t-test was used to analyze the data (NS, not significant; *p < 0.05; **p < 0.01; ***p < 0.001).

**Supplemental Figure 6. ADEs promote ovarian cancer metastasis in vivo.** Mice bearing SKOV3-luc orthotopic xenotransplants were injected intraperitoneally with #27 ADEs or PBS. (a) Bioluminescence images of abdominal metastasis in mice bearing SKOV3-luc orthotopic ovarian xenotransplants. (b) Growth curve of SKOV3-luc tumors based on quantitative radiance values from IVIS imaging. (c) Bioluminescence images of metastases on abdominal organs. The organs arranged clockwise from the top are the liver, spleen, kidney, pancreas and omentum. (d) Metastases on abdominal organs and ovarian primary tumors. Black arrow, ovarian primary tumors; red arrow, metastases on abdominal organs. (e) Statistical table showing the numbers of abdominal organs with metastasis. (f) Representative immunohistochemical images of the expression of E-cadherin, N-cadherin and Vimentin in orthotopic tumor tissues. Scale bar, 20 μm. (g) Diagram showing the calculated percentages of Ki67 and mean densities of E-cadherin, N-cadherin and Vimentin in the two groups (*p < 0.05; **p < 0.01; ***p < 0.001).

**Supplemental Figure 7. Identification of primary tumor cells.** (a) Representative images of cell morphology of T1 and T2. Scale bar, 100 μm. (b) Flow cytometric analysis of EpCAM in T1 and T2. Experiments were performed in triplicate. (c) Western blot analysis of the expression of PAX8, cytokeratin-8, E-cadherin, α-SMA in T1 and T2 are shown. (d) Immunocytochemistry of primary tumor cells to detect the expression of PAX8, E-cadherin, cytokeratin 8, calretinin and cytokeratin 8. Representative images are shown. Scale bar, 100 μm.

**Supplemental Figure 8. Morphologic changes in ovarian cancer cells after miR-6780b-5p regulation.** (a) Representative images of cell morphology of A2780, CAOV3, ES2, and SKOV3 cells. Scale bar, 100 μm.

**Supplemental Experimental Procedures**

### 1. NanoSight analysis (NTA) of ADEs #7 and #27.

NTA analysis of ADEs #7 and #27 were performed in a Zetasizer Ultra instrument (Malvern). The samples were added to the instrument to analyze the diameter and concentration of exosomes.

### 2. Flow cytometric analysis of ADEs #7 and #27.

Flow cytometric analysis of the surface marker of ADEs #7 and #27 was conducted in a BD Accuri C6 flow cytometer (BD Biosciences) according to the device instructions. CD63-Antibody-FITC (Biosciences, USA) and CD81-Antibody-PE (Biosciences, USA) were used to analyze the surface markers CD63 and CD81.

### 3.Immunohistochemical analysis of cell pellets derived from ascites

The collected ascites was centrifuged at 300 × g for 10 min, and cell pellets were acquired and subsequently fixed in 4% paraformal-dehyde for 1 h and embedded in paraffin. Hematoxylin and eosin (HE) staining and immunohistochemistry assays were performed. The slides were incubated with primary antibodies specific for positive markers of epithelial ovarian cancer [anti-PAX8 (Proteintech, USA), anti-E-cadherin (CST, USA), and anti-cytokeratin 8 (Abcam, USA)], a positive marker of mesenchymal cells [anti-vimentin (Abcam, USA)], a positive marker of monocytes and macrophages [anti-CD68 (protientech, USA)] and a positive marker of CAFs [anti-α-SMA (Abcam, USA)] at 4 °C overnight. Then, the cells were incubated with a biotinylated secondary antibody. Normal rabbit IgG was used as a negative control. Finally, the cells were immunostained with diaminobenzidine (DAB). Positive expression was defined as the appearance of brown-yellow granules. PBS substitution for the primary antibody served as the blank control.

### 4. Isolation and culture of primary ovarian cancer associated fibroblasts (CAFs).

Primary CAFs were extracted from T1 using the method described previously[^1^](#_ENREF_1) and cultured in DMEM/F12 medium supplemented with 10% fetal bovine serum (FBS, Gibco). All procedures were conducted with the approval of the Ethics Committee of Tongji Medical College, Huazhong University of Science and Technology. Patient consent was obtained before the start of the study.

### 5. Flow cytometric analysis of primary tumor cells.

Flow cytometric analysis of the epithelial cell marker EpCAM was conducted as described previously[^2^](#_ENREF_2) using an APC-conjugated anti-EpCAM antibody (Biolegend, USA) in an LSR flow cytometer (BD Biosciences, USA), and the data were analyzed using ModFit LT software (Verity Software House, USA).

### 6. Immunocytochemistry of primary tumor cells.

Primary tumor cells were fixed with 4% paraformaldehyde for 30 min at room temperature and used for immunocytochemistry. The cells were incubated with antibodies specific for positive markers of epithelial ovarian cancer [anti-PAX8, anti-E-cadherin (CST, USA), and anti-cytokeratin 8 (Abcam, USA)], a negative marker of ovarian cancer [anti-calretinin (Abcam, USA)], and a positive marker of CAFs [anti-α-SMA (Abcam, USA)] at 4 °C overnight. Then, the cells were incubated with a biotinylated secondary antibody. Normal rabbit IgG was used as a negative control. Finally, the cells were immunostained with diaminobenzidine (DAB). Positive expression was defined as the appearance of brown-yellow granules.

**Supplemental References**

1 Cai, J. et al. Fibroblasts in omentum activated by tumor cells promote ovarian cancer growth, adhesion and invasiveness. Carcinogenesis 33, 20-29, (2011).

2 Tang, H., Chu, Y., Huang, Z., Cai, J. & Wang, Z. The metastatic phenotype shift toward myofibroblast of adipose-derived mesenchymal stem cells promotes ovarian cancer progression. Carcinogenesis 41, 182-193, (2020).
