# Supplementary figures and images for: Exosomes in ovarian cancer ascites promote epithelial–mesenchymal transition of ovarian cancer cells by delivery of miR-6780b-5p
Source: Cell Death Dis. 2021 Feb 24;12(2):210. doi: 10.1038/s41419-021-03490-5 (PMC7904844; doi:10.1038/s41419-021-03490-5)

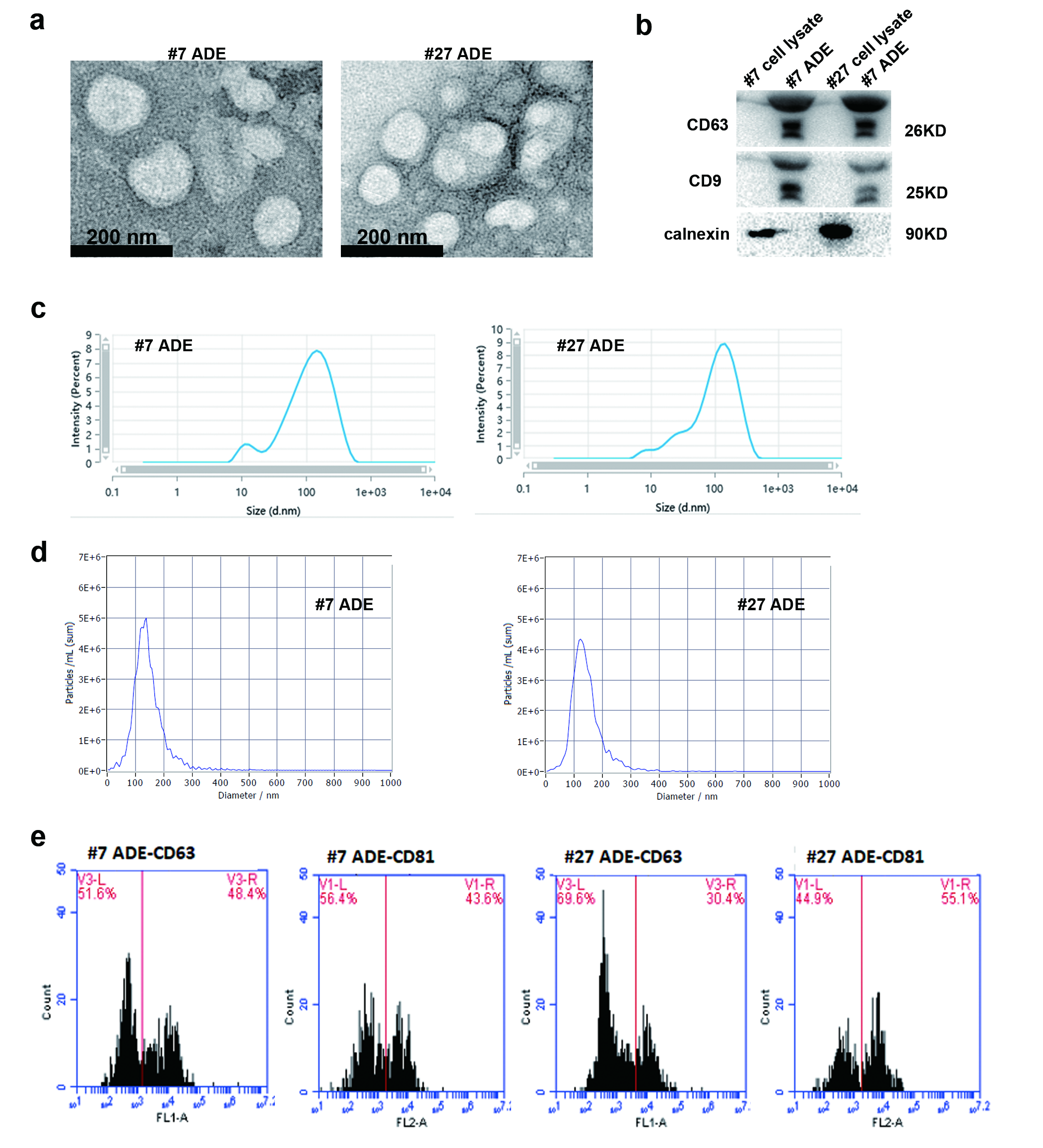

Supplement: Supplementary file 2 — Suplemental Figure 1 [file 41419_2021_3490_MOESM2_ESM.tif]

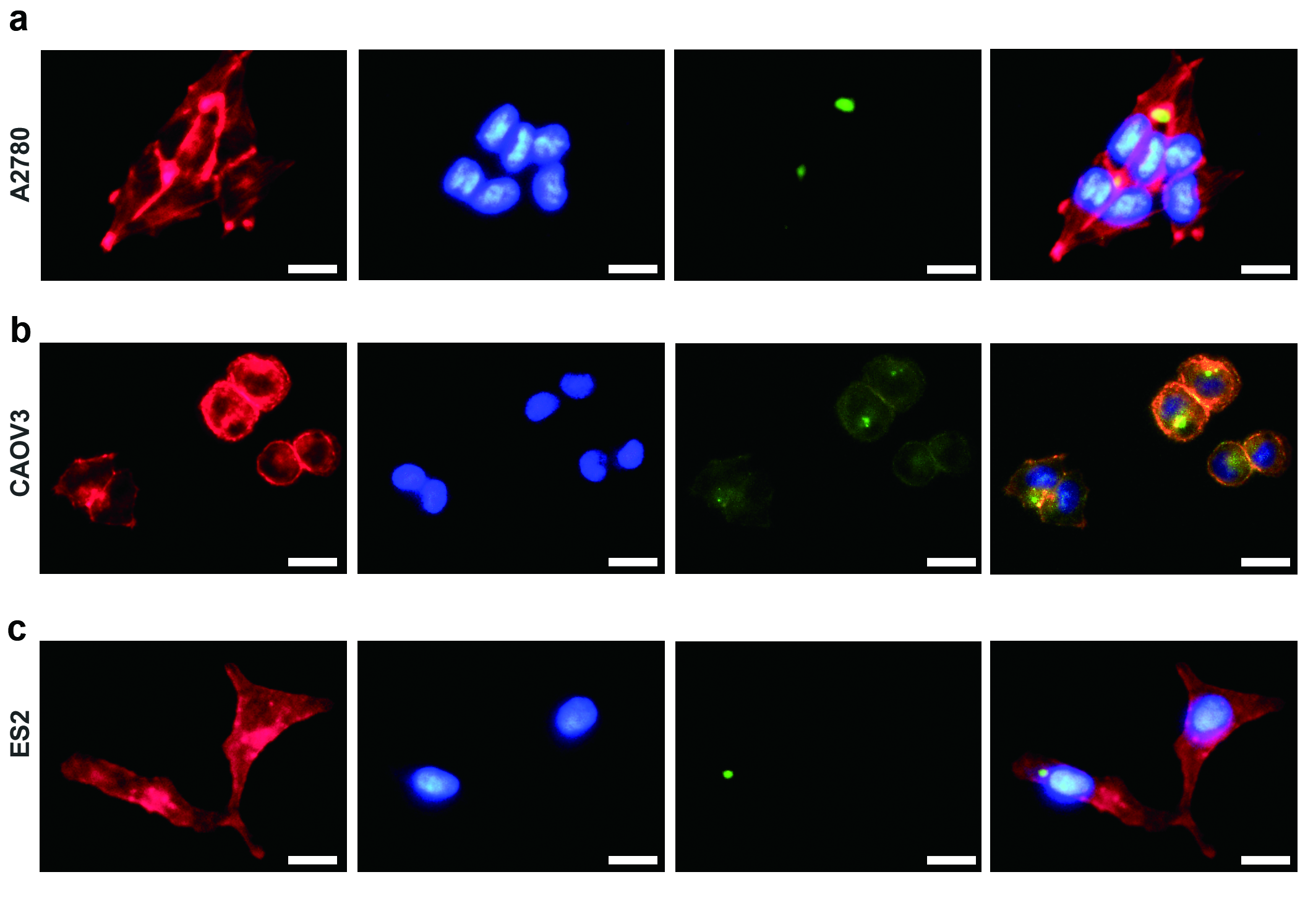

Supplement: Supplementary file 3 — Suplemental Figure 2 [file 41419_2021_3490_MOESM3_ESM.tif]

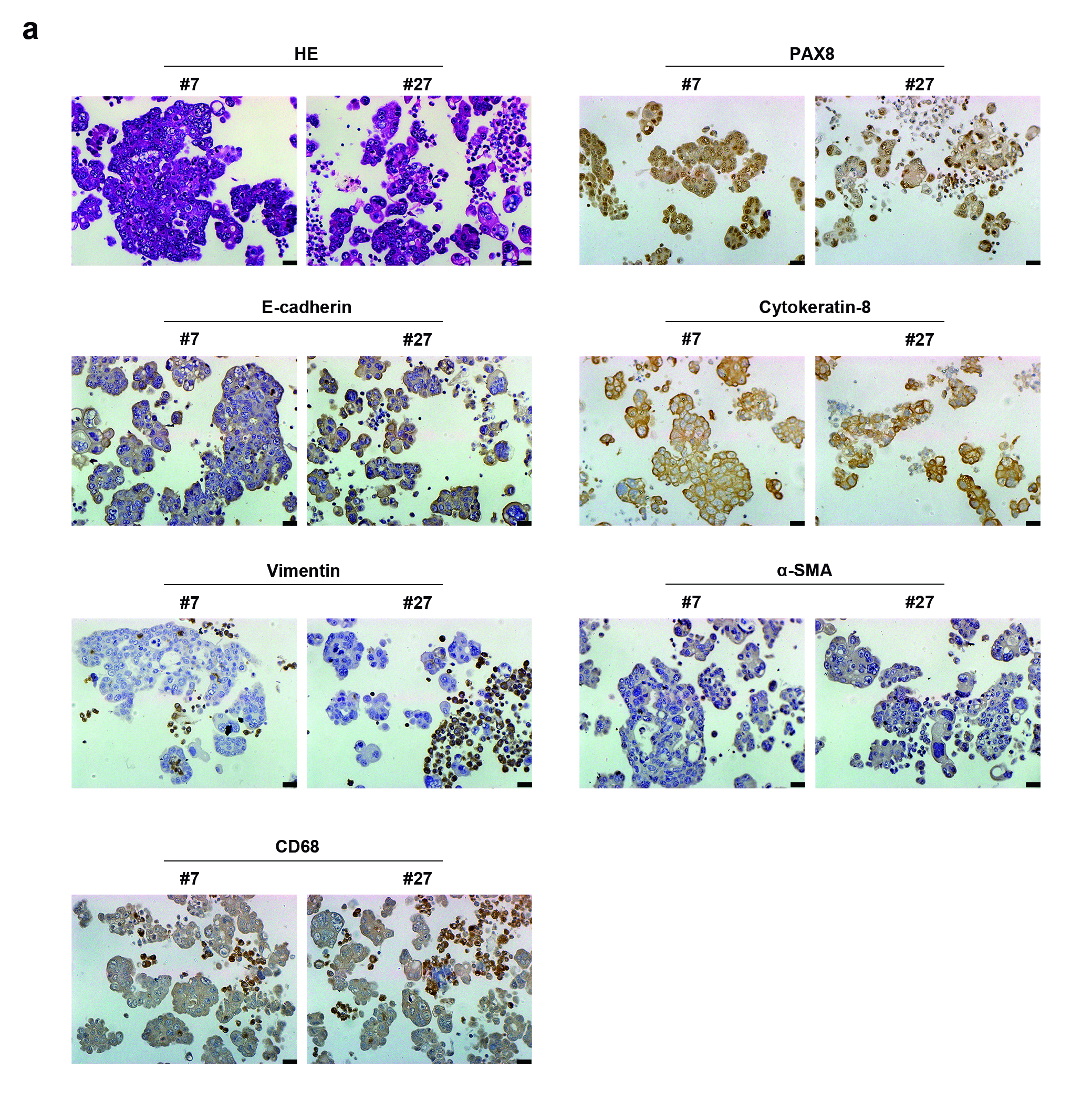

Supplement: Supplementary file 4 — Suplemental Figure 3 [file 41419_2021_3490_MOESM4_ESM.tif]

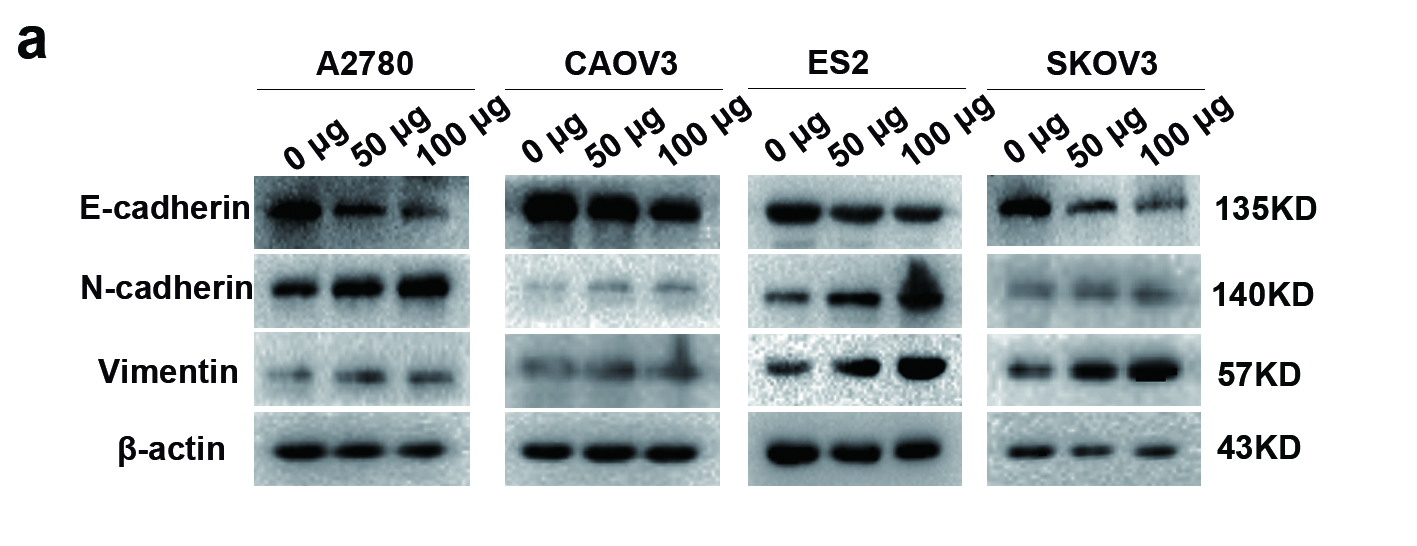

Supplement: Supplementary file 5 — Suplemental Figure 4 [file 41419_2021_3490_MOESM5_ESM.tif]

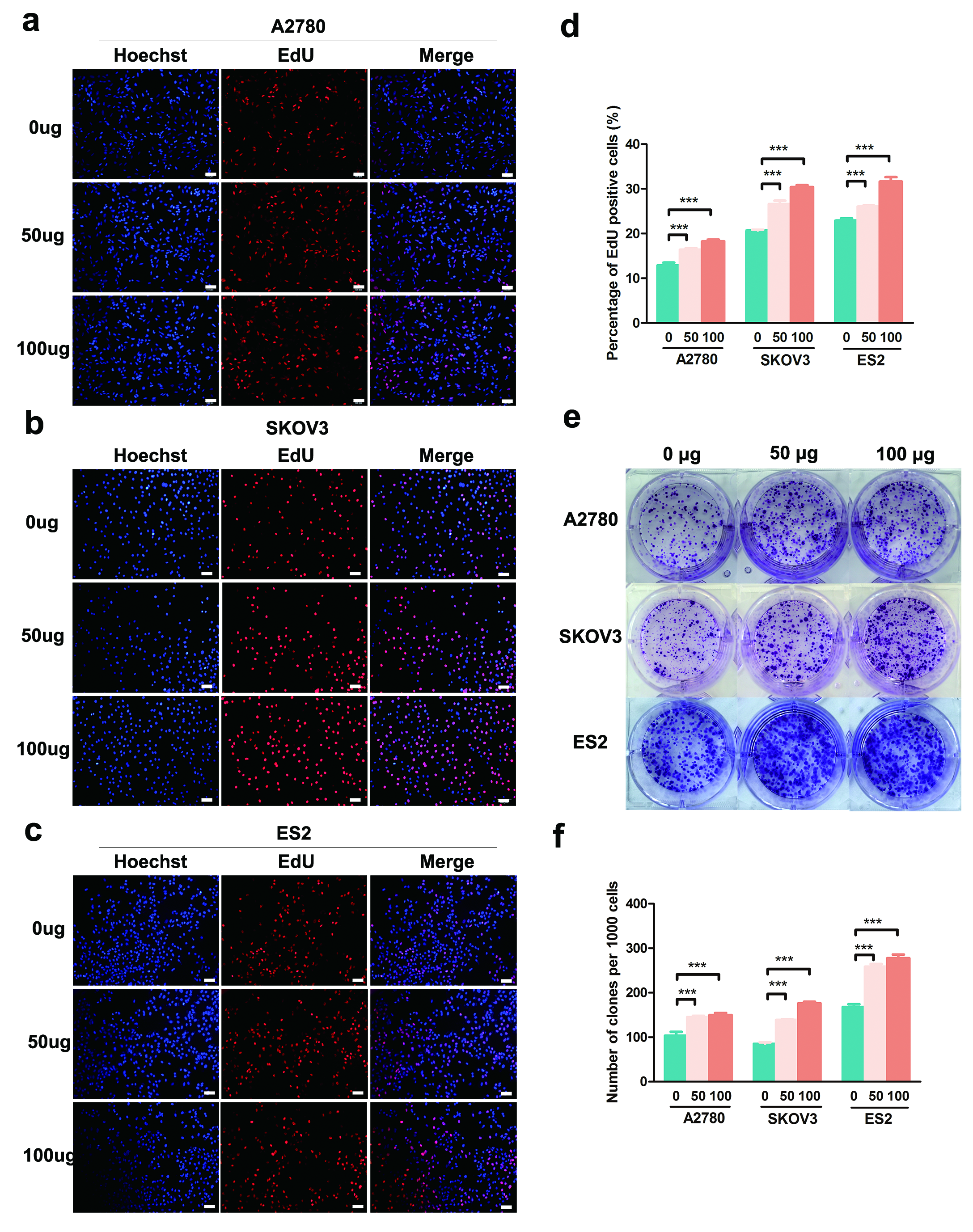

Supplement: Supplementary file 6 — Suplemental Figure 5 [file 41419_2021_3490_MOESM6_ESM.tif]

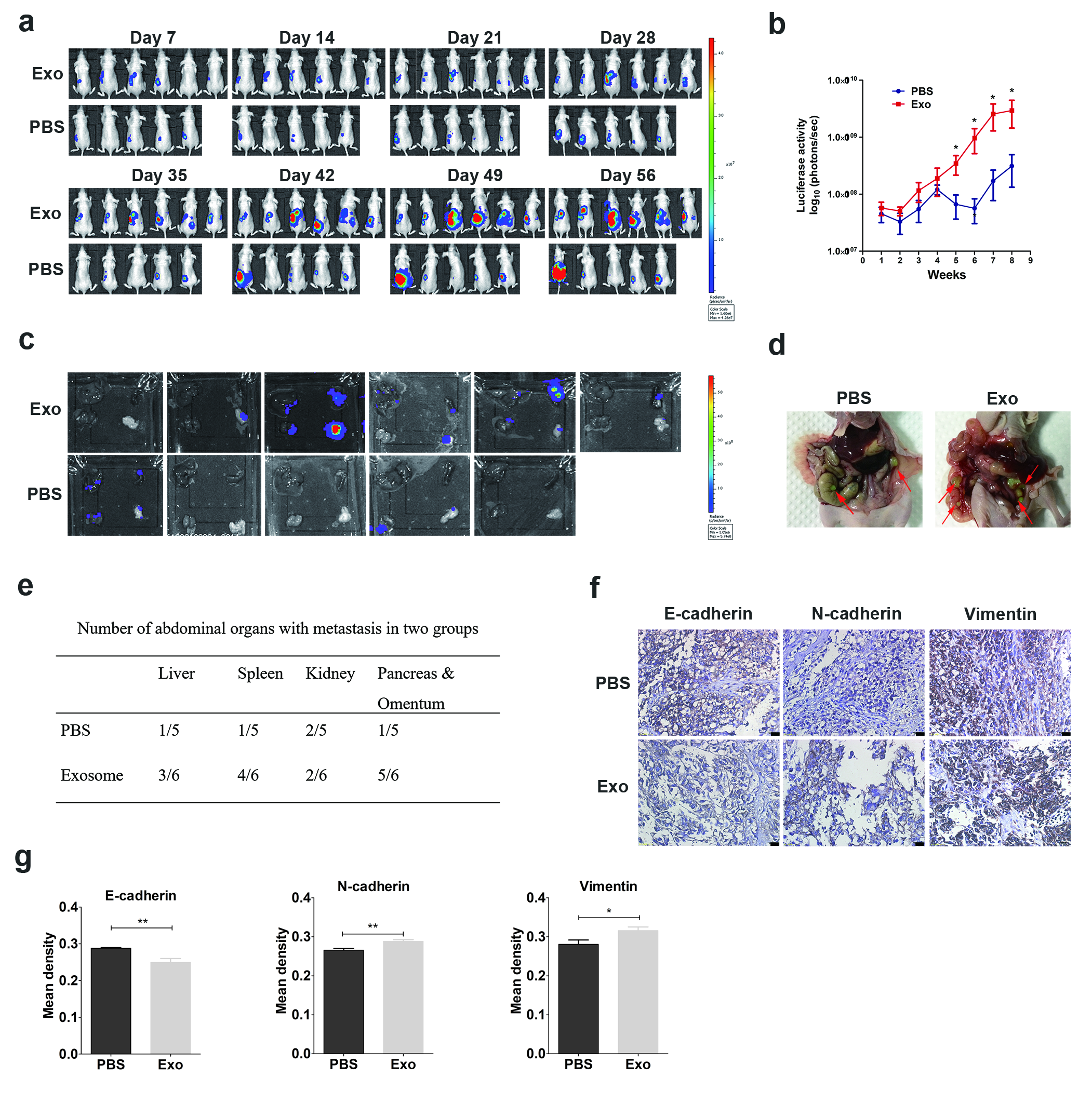

Supplement: Supplementary file 7 — Suplemental Figure 6 [file 41419_2021_3490_MOESM7_ESM.tif]

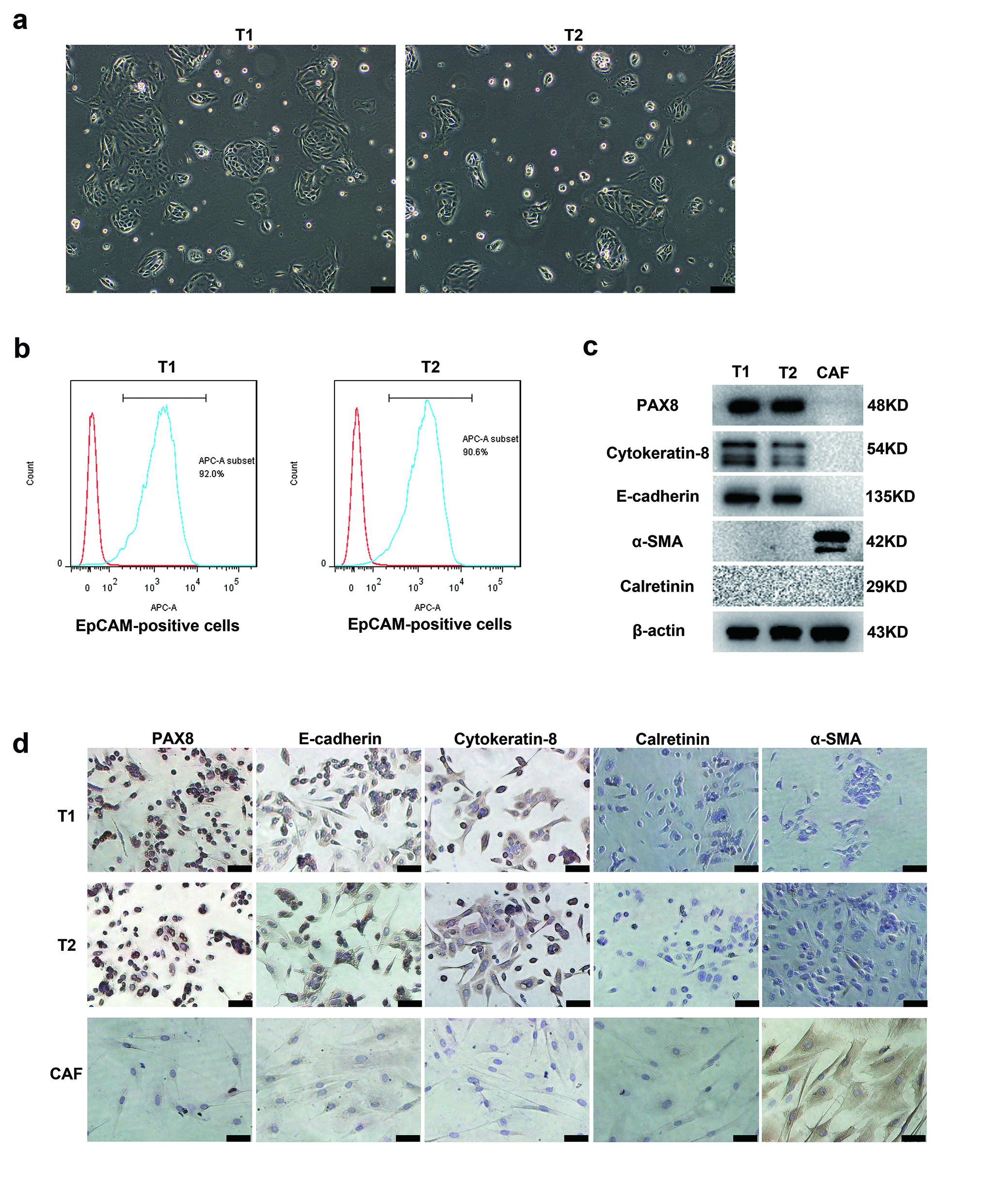

Supplement: Supplementary file 8 — Suplemental Figure 7 [file 41419_2021_3490_MOESM8_ESM.tif]

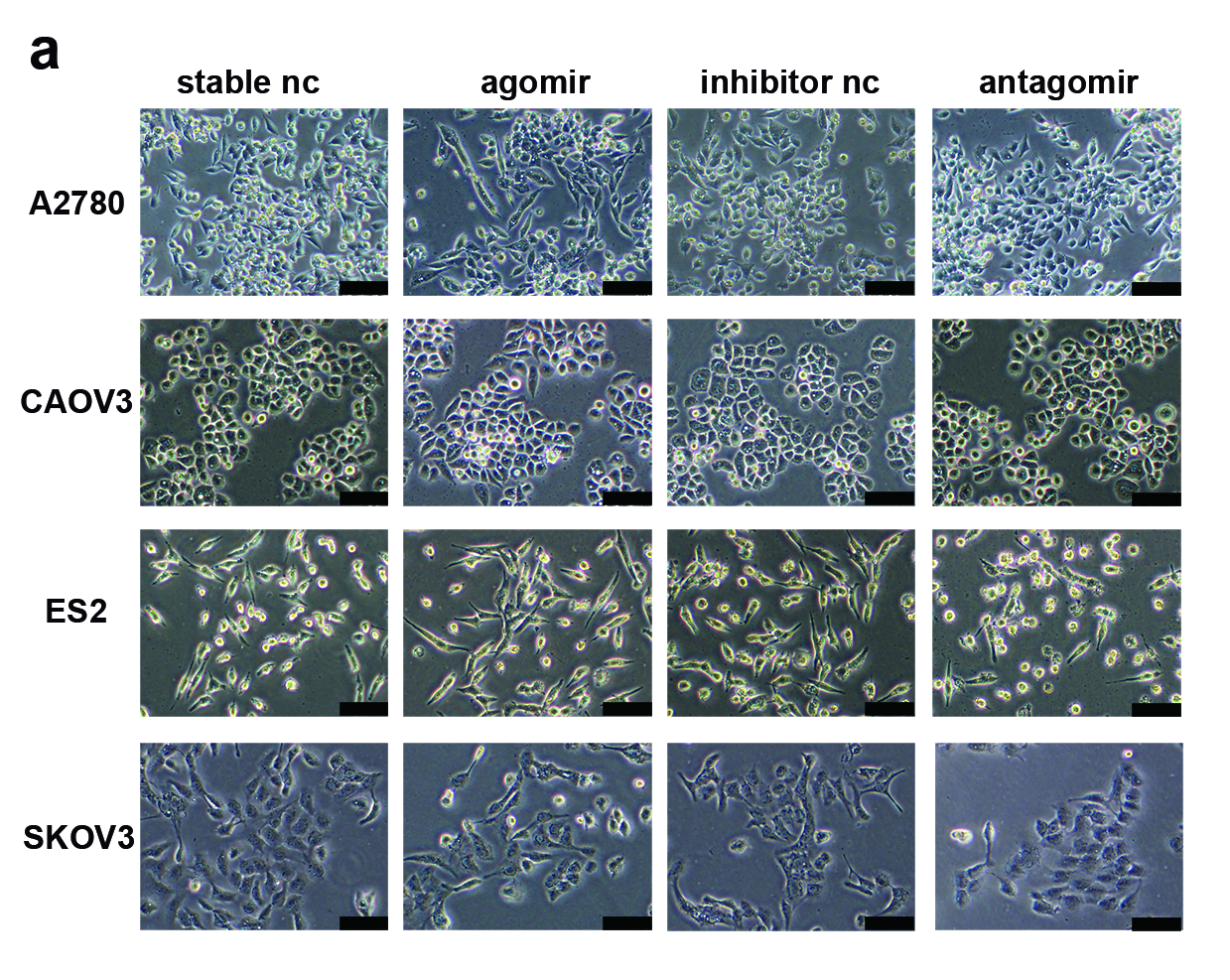

Supplement: Supplementary file 9 — Suplemental Figure 8 [file 41419_2021_3490_MOESM9_ESM.tif]
